# Appropriate sampling methods and statistics can tell apart fraud from pesticide drift in organic farming

**DOI:** 10.1038/s41598-021-93624-8

**Published:** 2021-07-20

**Authors:** Albrecht Benzing, Hans-Peter Piepho, Waqas Ahmed Malik, Maria R. Finckh, Manuel Mittelhammer, Dominic Strempel, Johannes Jaschik, Jochen Neuendorff, Liliana Guamán, José Mancheno, Luis Melo, Omar Pavón, Roberto Cangahuamín, Juan-Carlos Ullauri

**Affiliations:** 1 CERES GmbH, Vorderhaslach 1, 91230 Happurg, Germany; 2 Biostatistics Unit, Institute of Crop Science, University of Hohenheim, 70593 Stuttgart, Germany; 3 Department of Ecological Crop Protection, University of Kassel, Nordbahnhofstr. 1a, 37213 Witzenhausen, Germany; 4 Eurofins Dr. Specht International GmbH, Am Neulaender Gewerbepark 2, 21079 Hamburg, Germany; 5 Gesellschaft für Ressourcenschutz (GfRS), Prinzenstr. 4, 37073 Göttingen, Germany

**Keywords:** Ecology, Environmental sciences

## Abstract

Pesticide residues are much lower in organic than in conventional food. The article summarizes the available residue data from the EU and the U.S. organic market. Differences between samples from several sources suggest that organic products are declared conventional, when they have residues—but the origin of the residues is not always investigated. A large number of samples are being tested by organic certifiers, but the sampling methods often do not allow to determine if such residues stem from prohibited pesticide use by organic farmers, from mixing organic with conventional products, from short-range spray-drift from neighbour farms, from the ubiquitous presence of such substances due to long-distance drift, or from other sources of contamination. Eight case studies from different crops and countries are used to demonstrate that sampling at different distances from possible sources of short-distance drift in most cases allows differentiating deliberate pesticide application by the organic farmer from drift. Datasets from 67 banana farms in Ecuador, where aerial fungicide spraying leads to a heavy drift problem, were subjected to statistical analysis. A linear discriminant function including four variables was identified for distinguishing under these conditions application from drift, with an accuracy of 93.3%.

## Introduction

### Pesticide residues in organic products

**Figure 1:**
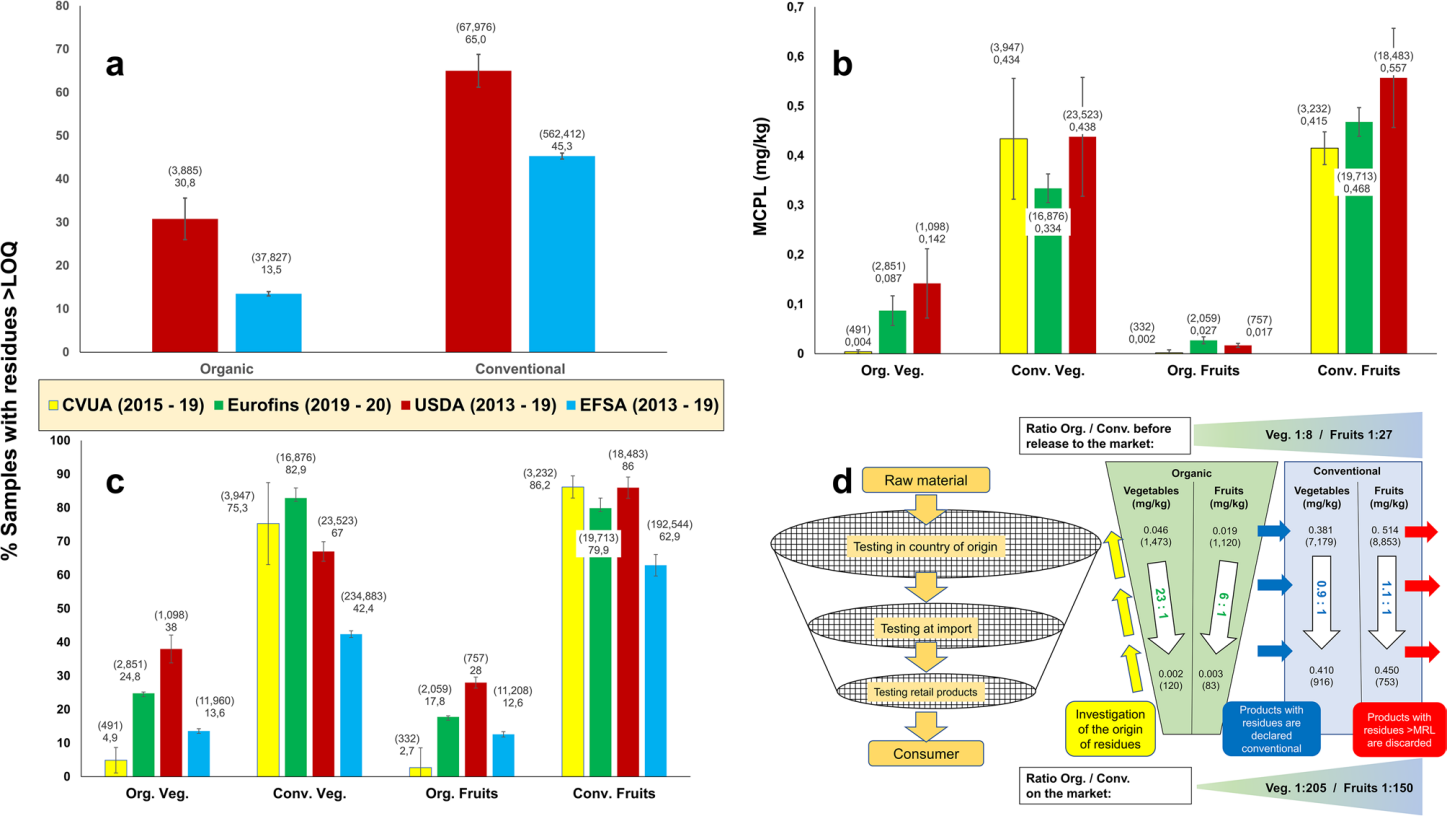
Pesticide residues in conventional and organic food in tests conducted by four organisations: EFSA (European Food Safety Authority) collects official data from all EU member states^3^, CVUA from one federal state in Germany^4^, USDA from government laboratories across the U.S.^5^, while Eurofins is a commercial laboratory in Germany. Figures in brackets represent the number of samples. The legend is valid for (**a**), (**b**) and (**c**). In order to increase the number of samples (represented in brackets) and thus their representativeness, figures from several years were grouped together, as available from each organisation. Black bars symbolise standard errors across years. (**a**) Shows the percentage of samples with residues above the limit of quantification (LOQ), for all types of food ( available from two organisations only). (**b**) Represents the mean cumulative pesticide load (MCPL) for fruits and vegetables (available from three organisations). (**c**) Similar to (**a**), but for fresh fruits and vegetables only (CVUA uses "above 0.01 mg/kg" instead of LOQ, but this is identical for most substances). The same datasets were used for (**b**) and (**c**). (**d**) Multi-layer sieving model for residue testing of fruits and vegetables in 2019, at different points of the organic supply chain. The data above the white arrows are from the commercial laboratory Eurofins, and mostly represent the situation **before** products are released to the market, while the figures below the white arrows are from CVUA, representing the situation **on** the market (both wholesale and retail). Ratios from "before market" to "on market" are shown in the white arrows. In this process, the MCPL remains in the same range for conventional products (blue rectangle to the right), while it is reduced massively for organic products (green trapezium in the centre). As a result of this sieving mechanism, residues in samples from the market are 150 and more times lower in organic than in conventional produce (trapezium at the bottom). This shows that the process represented by the blue arrows works fairly well—which is not always the case for the investigation of the origin of such residues, symbolised by the yellow arrows.

Non-use of synthetic pesticides is a major characteristic of organic farming, with the objectives of protecting (a) the environment, (b) consumer health, and (c) farm worker health. In consumer studies, "no chemical pesticides" is usually mentioned as one of the most important criteria for buying organic food^1,2^. These consumer expectations are mostly met in what is referred to in objective (b). Both European and U.S. sources consistently found the percentage of samples with residues of pesticides above the limit of quantification (> LOQ) to be much lower in organic than in conventional food (Fig. 1a, see also Supplementary Fig. 1). This is especially true when it comes to fresh fruits and vegetables (Fig. 1c), which are known to be the most critical food groups in terms of pesticide residues^3^. It is elucidating, however, to not only look at the number of samples with an (unknown) level of residues > LOQ, but to quantify the residues found per sample. In many cases, more than one substance is found in a sample, therefore one meaningful indicator is the mean cumulative pesticide load per sample (MCPL, see Supplementary Table 1). This is represented in Fig. 1b for three out of the four datasets. The food authority CVUA (Chemisches- und Veterinäruntersuchungsamt) in Baden-Württemberg, Germany, has been comparing pesticide residues between organic and conventional food since 2002. In 2019, on average the residues in organic produce were more than 150 times lower than in the corresponding conventional products^4^ (Ratio Org./Conv., bottom of Fig. 1d). The USDA (U.S. Department of Agriculture) numbers tend to be higher than the European ones, both in percentages (Fig. 1a,c) and in MCPL (Fig. 1b). One reason for this is probably USDA's risk-oriented sampling approach, in which some highly contaminated commodities are over-represented, as compared to their importance in most people's diet (Supplementary Table 2, column C). If we correct this possible bias by assuming that every commodity would have been sampled with the same frequency, the MCPL across all commodities is cut by 40% (Supplementary Table 2, last row). Different LOQs and numbers of analytes covered by USDA on one hand, and different European laboratories on the other hand, also make comparison difficult.

### Organic businesses' testing strategies

Unfortunately, the generally good news for consumers with respect to objective (b) does not always mean that objectives (a) and (c) are also met. With the steady growth of the organic market and globalisation of supply chains, integrity of the system is often at stake. Organic products mostly fetch higher prices, and therefore also attract fraud^6,7^. Since pesticide residues are easily detectable parameters, often indicating non-compliance with organic production rules, many organic businesses test each batch for such residues, before placing it on the market. Positive results should then lead to an investigation of the origin of the found residues: Did an organic farmer spray? Do the residues come from drift, from ubiquitous contamination, or from (avoidable or unavoidable) contamination during processing, transport, storage? Were organic and conventional products mixed at some point of the supply chain—or is somebody simply labelling conventional products as "organic"? The idea behind this is depicted in Fig. 1d. The filter process as such, and the exclusion of contaminated batches from the organic market, as represented by the blue and red arrows, often work well. Thus, there are remarkably lower average amounts of residues after undergoing this filtering process. Residues in organic produce reported from the market were reduced by 6 and 23 times in fruits and vegetables, respectively, compared to the levels reported by the commercial laboratory, which represent mostly pre-market samples, while the values for conventional samples remained in the same range. This shows that market actors often remove problematic batches by declaring them conventional. In Supplementary Tables 3 and 4 we provide further explanations why the datasets "before release to the market" and "on the market" in Fig. 1d are comparable.

We do not have test results from a commercial laboratory in the U.S. that could be compared to Eurofins data. But, as opposed to the other sources of information, the USDA database identifies the country of origin of each sample. Anybody working in international organic certification would expect residues in imported food to be higher than in domestic products, because fraud is more widespread when the distance is bigger between producers on the one hand, and consumers and the competent authorities on the other. The U.S. data, however, suggest the opposite trend: Not only at the aggregate level, but also for most individual commodities, the MCPL is lower in imported than in domestic products (Supplementary Table 2, columns J and K). The reason is probably that samples are tested before signing purchase contracts, and products rejected or bought as conventional, if they do not comply with the expectations.

This is good quality control practice—the problem is that the information about the "downgrading" of organic products to conventional is not always reaching the certification bodies (CBs), thus impeding the investigation of the origin of residues and the exclusion of fraudulent actors from the market (yellow arrows in Fig. 1d). It is in the nature of things that these processes are not publicly known and therefore cannot be quantified, but in Supplementary Fig. 2 we present anecdotic evidence, which also suggests that for some market actors the definition of "organic" is limited to "free of pesticide residues".

### Certifiers' testing strategies

The two most important markets for organic food are the EU, where the "organic" label is legally governed by an EU Regulation, and the USA, where the corresponding rule is the National Organic Program (NOP). Although they have different approaches on how to deal with spray-drift and with residues (Supplementary Table 5), both regulations require CBs to take samples from at least 5% of their clients every year. A large amount of data is being generated through this mechanism, but the sampling procedures and interpretation of results often do not allow deriving clear results. A recent unpublished BSc thesis at the University of Kassel revealed that 80% of the samples by CBs in ten EU member countries are taken of final products, but only 20% from the field or during the production process. This suggests that not only for market actors, but also for many CBs, the purpose of sampling and testing is limited to ensuring that food sold on the market with an organic claim, is free of pesticide residues, without digging deeper to find the origin of contamination.

The differentiation between active use and non-intentional contamination is difficult, if only final products are tested. Plant (mainly leaf) samples from the field have several advantages in this regard: (a) Often, there is a long time span between pesticide application and harvest. Because of dissipation of the residues, nothing or only traces may be found in the final product (Supplementary Table 6). Field samples can be taken during or shortly after a suspected pesticide application, so that the dissipation effect is reduced and residues are found even for substances with a short half-life. (b) Leaves have a surface/weight ratio between 10 and 118 cm^2^/g^8^, whereas for fruits this ratio is between 0.6 and 2.2^9^, and for seeds between 2 and 10 cm^2^/g only^10–12^. Residues in leaves are therefore normally higher than in seeds, fruits or roots, which makes interpretation of test results easier. (c) Field sampling allows taking separate samples from centre and margin of the field, as explained below in more detail.

Unfortunately, if CBs take field samples at all, they often take them **only** from field margins^13,14^ ("let's see if there is a drift problem"). Positive results are then attributed to spray-drift, and farmers are required to establish buffers—without even considering the possibility of residues originating from an application by the organic farmer. Such procedures open the door for fraudulent use of pesticides by organic farmers.

Other CBs have established so-called "action levels", below which they consider the presence of residues in organic products to be the result of ubiquitous environmental contamination, with no need to investigate their origin^13^. While such thresholds may be necessary for specific cases (see below concerning the banana industry), using this approach as a general procedure disregards not only the spatial distribution, but also temporal dynamics of pesticides in plant tissue. As opposed to soil, half-lives in plant tissue exposed to UV radiation and weather, are relatively short for most modern pesticides^15^. A residue level of 0.02 mg/kg, used by some CBs as "action level", is typically reached one to two months after the application of a pesticide, in some cases even after only five days (Supplementary Table 6).

The time that has elapsed since an application, however, is unknown in most cases. Spraying records kept by conventional neighbours are normally not part of the inspection. In case of suspicious test results in samples from the organic farm, such records may sometimes be accessed as part of a follow-up investigation, but at that point the organic farmer may have asked the neighbour to manipulate the records. And if the organic farmer has sprayed, he or she obviously tries to hide this fact. This situation makes interpretation of low levels of residues found in samples from organic fields even more challenging, and increases the importance of being able to differentiate application from drift through other methods.

### Two forms of spray-drift

**Figure 2:**
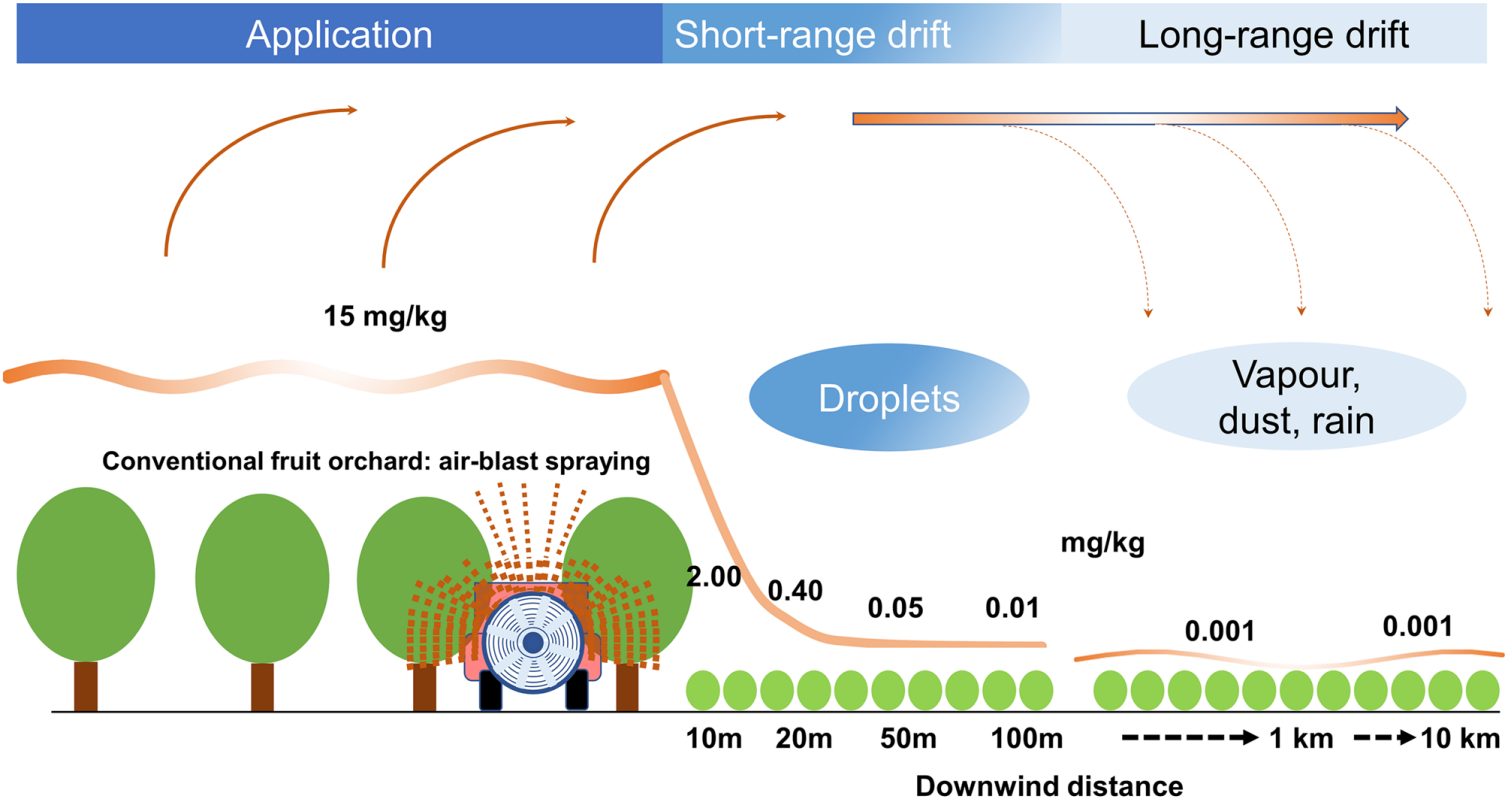
Simplified model of short-range vs. long-range drift originating from air-blast spraying in a fruit orchard. The specific values for pesticide concentrations (mg/kg) expected for different downwind distances from the orchard can vary by a factor 10 or more, depending on the applied substance, dose, weather conditions, vegetation, etc., but the graph provides an approximate estimate of the ratios that can be expected. In the case presented here, pesticide concentration in fruit leaves immediately after the application is 15 mg/kg. In the area of short-range direct drift, deposit decreases exponentially, so that at 100 m distance, we can expect to find only 0.01 mg/kg. At further distances, deposits are often below this level.

Over the past decades, a distinction has been made between short distance **primary** spray-drift **during** the application, and long distance **secondary** spray-drift occurring **after** the application^16^. The latter was attributed to evaporation and considered to play a role only for pesticides with high vapour pressure^17^. On the one hand, recent studies have shown that evaporation and long-distance transport can already play a role **during**, not only after application^18^. On the other hand, long-distance transport has been found to be linked not only to evaporation. Pesticides adherent to dust from wind erosion can contaminate large areas^19^. In the present context, we use the terms **short-range** and **long-range** drift, instead of primary and secondary drift (Fig. 2).

### Long-range drift

Long-range drift is so far poorly understood, can lead to (normally very low) residues at distances as far as thousands of km^19^, and happens in the form of vapour or molecules adhering to dust. The main factors influencing long-range drift are vapour pressure of the pesticide, capacity of adherence to dust, incidence of wind erosion, and temperature inversion in the atmosphere^17^. Long-range pesticide drift has recently received more attention^21–25^. Examples have been used in the context of organic certification for supporting the argument of ubiquity of pesticides, linked to the assumption that low- or even medium-level residues in organic products are often derived from their omnipresence in the environment^26,27^.

Cases from Brazil (endosulfan in soybeans), Montana (USA) and Saskatchewan (Canada) (glyphosate in khorasan wheat) and Germany (pendimethalin and prosulfocarb in different crops) have been quoted to demonstrate the ubiquity of pesticides^27^. None of these case studies, however, provides solid evidence for the assumption that long-distance transport of pesticides leads to residues in organic food above the level of, say, 0.01 to 0.03 mg/kg. The problem of the herbicides pendimethalin and prosulfocarb being subject to long-distance drift because of their high vapour pressure, has been known for a long time^28^, but this phenomenon cannot be extrapolated to other substances. Even for these herbicides, there is no evidence that residues at larger distances could be above the indicated levels. Across 15 vegetation samples from nature reserves in Germany, on average, 0.009 mg/kg pendimethalin and 0.004 mg/kg prosulfocarb were found^29^. Exceptions may exist, e.g., when pesticide applications are followed by heavy wind erosion, as seems to be the case in some of the North American wheat growing areas, where glyphosate is used for cereal desiccation shortly before harvest.

**Figure 3:**
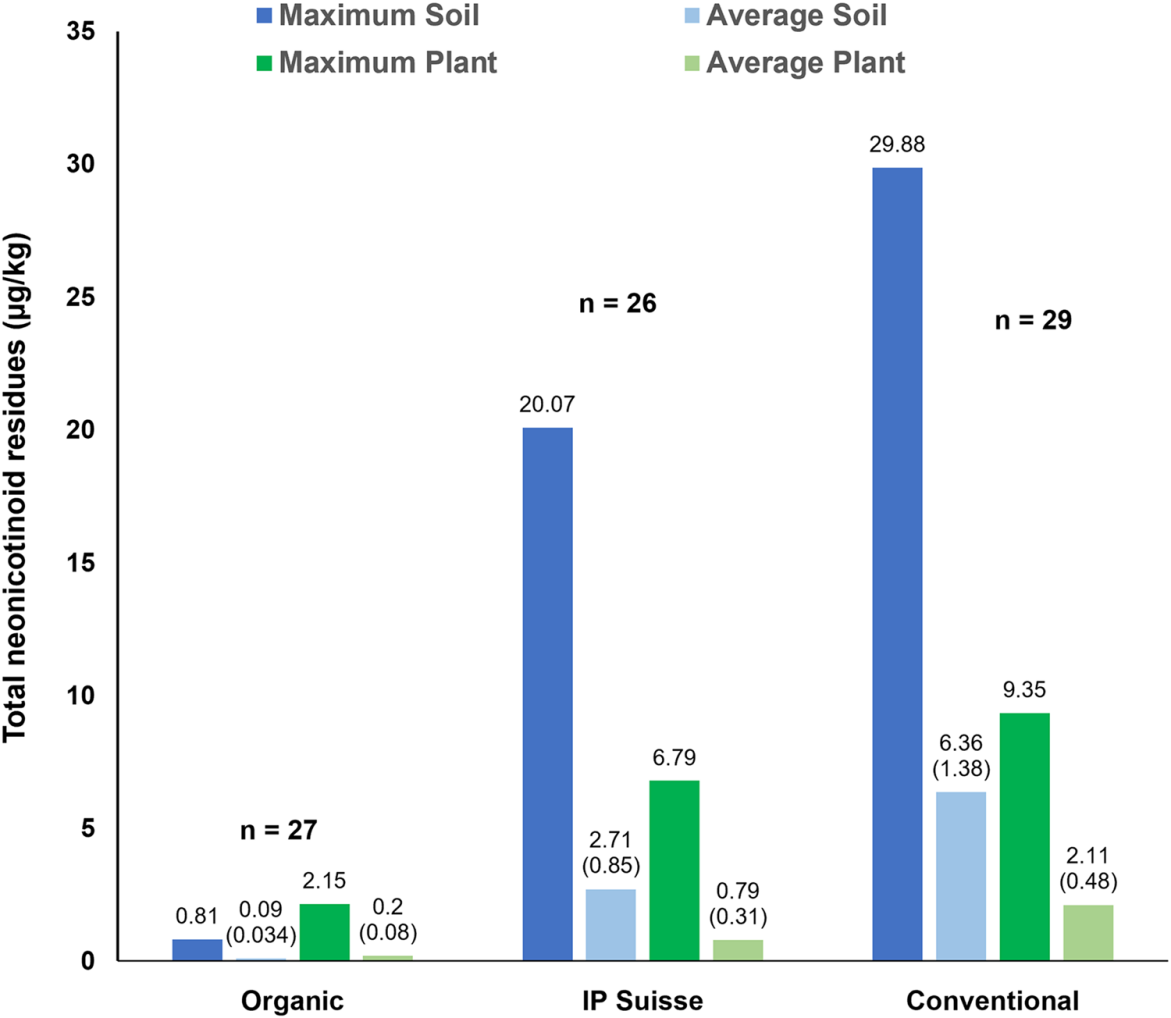
Maximum and average residues of neonicotinoid insecticides in soil and plant samples from organic farms, integrated crop production ("IP Suisse": this program involves reduced pesticide application) and conventional farms in Switzerland. The figures represent the sums of acetamiprid, chlothianidin, imidacloprid, thiacloprid and thiamethoxan. Figures in brackets represent standard errors.(Data from Humann-Guilleminot et al.^30^).

In a survey in Switzerland^30^, neonicotinoid residues were found in 93% of plant samples from organic farms (as compared to 100% of samples from conventional farms), thus supporting the ubiquity suspicion. But there were substantial quantitative differences between organic and conventional farms (Fig. 3). The average sum of neonicotinoid residues in plant and soil samples from organic farms was lower by a factor of 11 than that of plant samples from conventional farms. For soil samples, this factor was as high as 71. Even the highest value for one single substance (imidacloprid) found in organic plants (2.13 µg/kg = 0.00213 mg/kg) would be below the limit of quantification (LOQ) used for this substance in most screenings (0.01 mg/kg).

In a study in Germany^29^, the MCPL in natural vegetation in five reference areas (average distance from arable fields >3 km) was 0.003 mg/kg, and in 15 nature conservation areas (average distance from arable fields 143 m) it was 0.006 mg/kg, but in three buffer zones (average distance 54 m) it was 5.4 mg/kg. To make figures comparable with other data in this article, we have subtracted the concentration of non-agricultural pesticides from the total amounts, and divided the numbers by a factor five, because the residues in this study refer to dry matter, while all the others use fresh matter. Although 5.4 mg/kg at 54 m distance is a disturbingly high value, the survey confirms that concentrations at larger distances do not exceed the "traces" level. The intention of this article is not to put in doubt the environmental damage caused by such traces. What we try to show is that the "ubiquity" argument may sometimes be hiding cases of fraudulent pesticide use by organic farmers.

### Short-range drift

As opposed to long-range drift, short-range drift is well understood, has its impact mainly in a range from 1 m up to a maximum of 1,000 m (for aerial spraying), happens in the form of droplets, and is not substance specific. The main factors influencing this form of drift are droplet size, windspeed, and height of the boom (nozzles) above soil^17,19,31–33^. The fact that long-range drift is poorly understood and leads to low concentrations of certain substances over wide areas, should not stop certification bodies (CBs) from using the available knowledge about short-range drift as a tool for assessing farmers' compliance with organic production rules. The dynamics of short-range spray-drift have been widely studied in the context of preventing liability problems due to herbicide damage, contamination of water bodies and natural habitats, and direct risks for human settlements^19,31–36^. Pesticide deposit decreases exponentially with increasing distance from the field on which the substance is applied. With a tractor boom sprayer, deposit at 25 m distance is expected to be only 1% of that in the target field. While distances are greater for air-blast or aerial spraying, the basic principle of exponential decrease is the same (Fig. 2 and Supplementary Fig. 3).

### Objectives

The objectives of our study are: (I) to demonstrate that appropriate field sampling methods can differentiate the effects of fraudulent pesticide application by the organic farmer, from the results of both short-range and long-range spray-drift, and (II) for the specific case of aerial fungicide spraying in the banana industry, identify appropriate variables, which allow us to interpret the test results correctly for the purpose of this differentiation.

## Materials and methods

### Field sampling methods to differentiate application from spray-drift

CERES (Certification of Environmental Standards) and GfRS (Gesellschaft für Ressourcenschutz) are private certification bodies conducting onsite inspections of thousands of organic farms every year: GfRS in Germany, CERES in more than 60 countries around the world. Copies of farm descriptions, inspection reports, sampling records and test results are kept in the digital archives of both organisations. To demonstrate the appropriateness of differentiated field sampling (objective I), in a first part of our study we selected from the CERES archive seven cases (Table 1), and one case from the GfRS files (N° 6 in Table 1). Criteria for the selection of these cases were to cover a diversity of crops, countries, farm sizes and chemical substances, as well as the entire range of conclusions from "the organic farm did not do anything wrong" to "the organic farm used prohibited pesticides and its certificate was withdrawn" through "the investigation did not lead to a clear conclusion".

**Table 1:** Eight case studies from nine different crops and seven different countries. Overview of substances and residue levels in margin and centre samples, specific conditions that were considered for decision making, and the final conclusion.

Country	Crop, type of sample	Farm	Substance	Type^a^	Residues (mg/kg)		Specific conditions	Conclusion
					Margin^b^	Centre^b^		
1. Chile	Apples: leaves	1	Captan	F	9.100	0.310	High level of spray-drift because of air-blast spraying on conventional neighbour farms Dist.100 m	Drift
			Acetamiprid	I	3.600	0.036		
			Fluopyram	F	0.120	0.010		
			Difenoconazole	F	0.110	–		
			Azoxystrobin	F	0.110	–		
			Spinosad^d^	I	0.092	0.200		
			Tebuconazole	F	0.037	Tr. < 0.020		
			Atrazine	H	0.022	0.016		
			Pyraclostrobin	F	0.011	–		
			Phosmet	I	Tr. < 0.020	–		
			Methidathion	I	Tr. < 0.010	Tr. < 0.010		
	Blueberries: leaves	2	Imidacloprid	I	0.150	1.800		Application
2. Togo	Soybeans: dry plants and weeds	1	Chlorpyrifos	I	–	0.023	Small fields, low spray-drift because farmers use manual knapsack sprayers. Dist.20–40 m	Probably application
			λ-Cyhalothrin	I	0.076	–		Drift
			Dichlobenil	H	0.005	Tr. < 0.005		Drift
		2	Deltamethrin	I	–	Tr. < 0.010		Drift
		3	Fipronil	I	0.120			Application^e^
3. Thailand	Rice: straw	1	Bifenthrin	I	0.011	0.013	Small fields, good buffers^f^, irrigation	Unclear
			Chlorpyrifos	I	0.005	0.007		Unclear
4. Ecuador	Cocoa: beans	1	2,4-D	H	0.018		Conventional banana farms with a high level of drift in the neighbourhood. Dist.100–300 m	NA^g^
	Cocoa: leaves		Glyphosate	H	Tr. < 0.010			Unclear
			Fenpropidin	F	0.026			Drift
			Pyrimethanil	F	0.010			Drift
	Cocoa: weeds		2,4-D	H	-	0.023		Application
			Glyphosate	H	0.023 0.021	0.011		Unclear^h^
			Chlorpyrifos	I	–	0.120		Drift
			Spiroxamine	F	–	0.019		Drift
			Fenpropidin	F	–	0.027		Drift
			Difenoconazole	F	–	0.014		Drift
5. Bulgaria	Oil bearing roses: leaves	1	Penconazole	F	(No border sample)	0.620	The inspector had been made believe that a risk of spray-drift did not exist, because conventional neighbour fields were semi-abandoned	Application
6. Germany	Vineyards: leaves	1	Folpet Dithiocarb.^i^	F	0.320 0.020	0.250 0.020	Small fields, very heavy drift from air-blast spraying, steep hill, samples taken during spraying season	Drift
		2	Folpet Dithiocarb	F	0.120 0.060	0.140 0.050		Drift
		3	Folpet Dithiocarb	F	0.160 –	0.230 –		Drift
		4	Folpet Dithiocarb	F	0.330 0.010	0.390 0.020		Drift
		5	Folpet Dithiocarb	F	0.120 0.040	0.140 0.020		Drift
		6	Folpet Dithiocarb	F	0.640 0.060	0.750 0.050		Drift
		7	Folpet Dithiocarb	F	0.250 0.210	0.220 0.150		Drift
		8	Folpet Dithiocarb	F	69.200 –	73.000 –		Application
7. Moldova	Walnuts: kernels	NA^g^	2,4–D	H	0.013–0.031		Wild or abandoned walnut trees in several areas, some of these close to cereal fields. Dist.100–400 m	NA^g^
		1	2,4-D	H	Tr. < 0.010	–		Drift
		2	2,4-D	H	Tr. < 0.010	–		Drift
		NA^g^	2,4-D	H	–			Postharvest mixing
8. Ecuador	Bananas: leaves	1	See Fig. 4a	F	See Fig. 4a		Very high level of spray-drift because of aerial fungicide^j^ spraying. Dist. 200 m	Application
		2	See Fig. 4b	F	See Fig. 4b			Drift

^a^F = Fungicide, H = Herbicide, I = Insecticide.

^b^"Tr.<" - traces below the indicated LOQ were found, but could not be quantified with the applied method.

^c^Dist.—Approximate distance between a possible source of drift and the centre of the organic field.

^d^Spinosad is an insecticide produced by micro-organisms, which is allowed in organic farming.

^e^Since there was no nearby conventional farm, only one sample was taken. Fipronil is mostly used for control of ectoparasite on animals, but can also be used for controlling termites.

^f^Buffers—the organic farm has hedgerows (living fences) protecting it from short-range drift.

^g^NA = Not applicable. The finding of residues in the cocoa beans and walnut kernels, respectively, imported into the EU, was the starting point of the investigation.

^h^Samples were taken from two field margins, therefore two different values are mentioned for these substances in the "margin" column.

^i^Dithiocarb. = Dithiocarbamates. A group of fungicides. The individual substances are not further differentiated by the used testing method.

^j^In addition to fungicides, conventional banana farmers also use herbicides and insecticides. These, however, are applied via motorised knapsack sprayers. Therefore, drift for these substances is far less than for fungicides. Insecticides are also used for impregnating the plastic bags covering the banana bunches.

**Figure 4:**
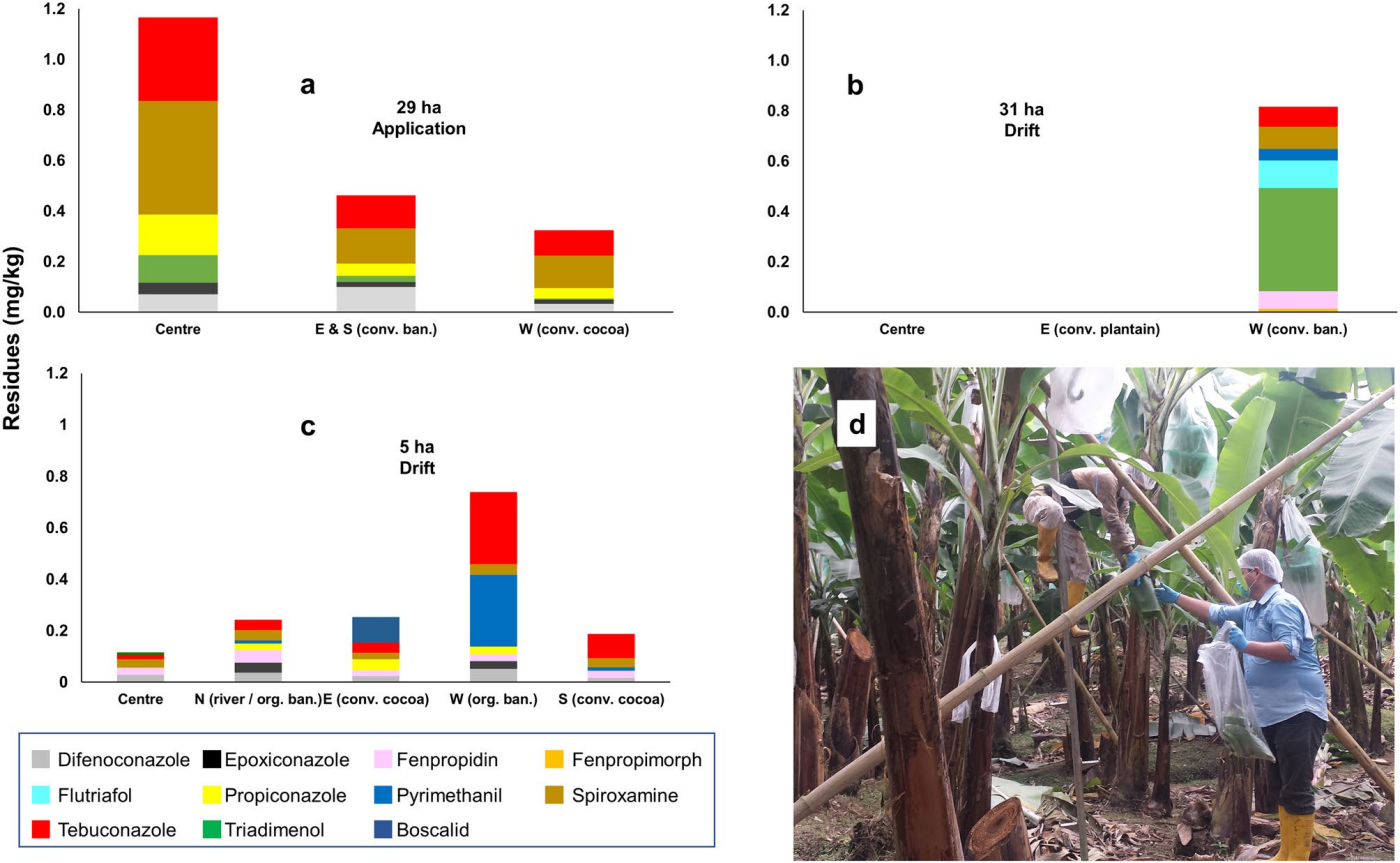
Residues of different fungicides found in leaf samples from three banana farms in Ecuador: (**a**) is a clear "application" case, (**b**) is a clear "drift" case (see also Supplementary Fig. 8). Also (**c**) is a "drift" case, but more complex because of the small size of the farm and the many different substances involved. Interestingly, the drift in (**c**) comes from the West (which is also the main direction of wind), where another organic banana farm (not certified by CERES) is located ("org. ban.", "conv. ban.", etc. refer to organic banana, conventional banana, cocoa and plantain farms as neighbours on each side; N, E, S, W to the cardinal points). (a) and (b) are cases from 2017, and are therefore not included in the statistical evaluation, while (**c**) represents case N° 49 (see also Fig. 5). (**d**) Sampling banana leaves. Photo by L. Guamán.

**Figure 5:**
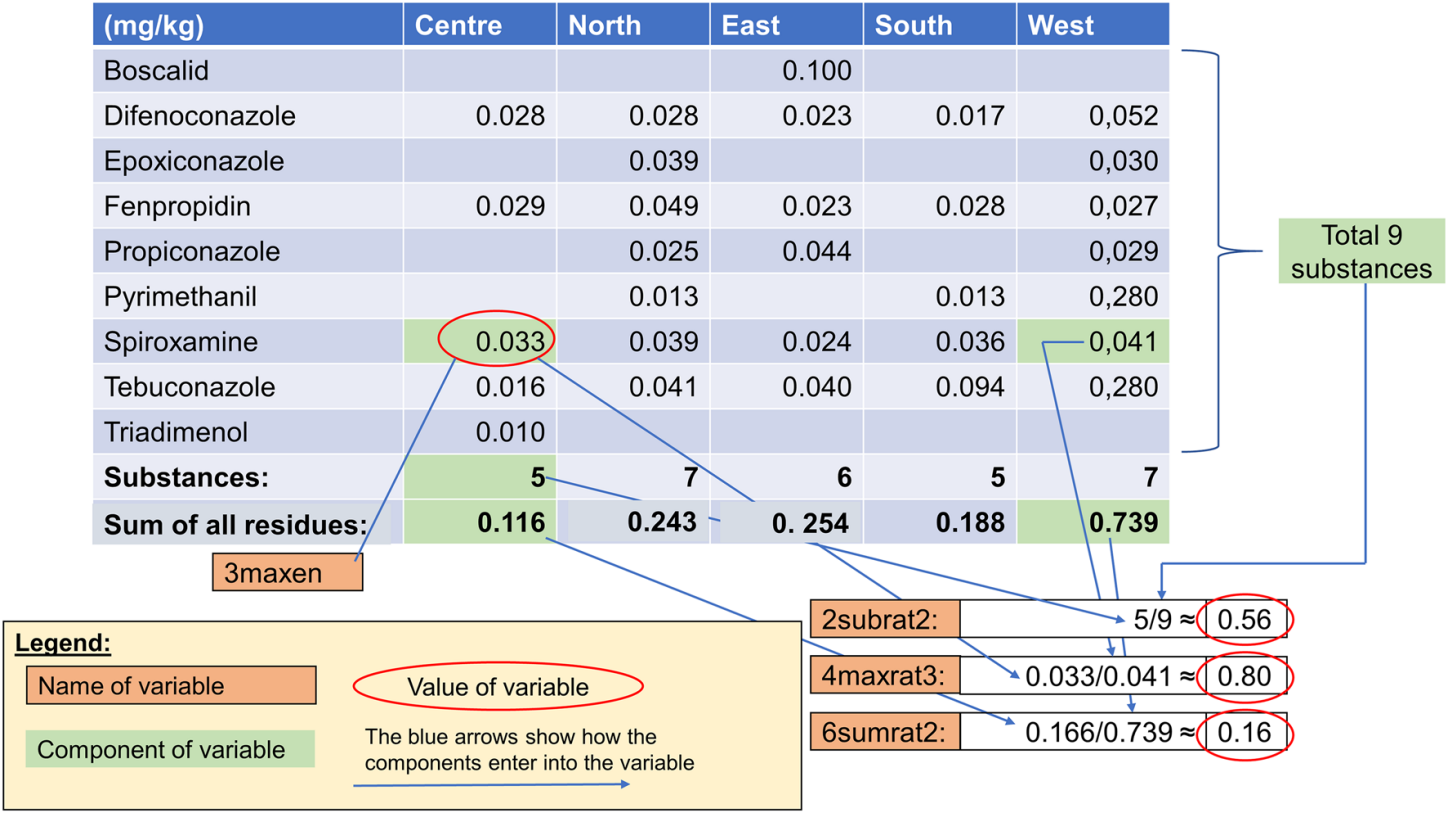
Raw data for case 49 (see also Fig. 4c), including an explanation of the four selected variables. A total of 5 fungicides were found in the centre, while in total 9 different fungicides were found in all samples, therefore the ratio of the two (called "2subrat2") is 5/9≈0.56. The value 0.033 mg/kg for spiroxamine is the highest figure out of the five residues found in the centre (here called "3maxcen"). We compare this to the highest value for spiroxamine among all samples, which is 0.041 mg/kg. The ratio of the two (called "4maxrat3") is 0.033/0.041≈0.80. The sum of all residues from the centre is 0.116 mg/kg, whereas the highest sum of residues from all samples is 0.739 mg/kg. The ratio of both, here called "6sumrat2", is 0.116/0.739≈0.16.

For differentiating drift from an application by the organic farmer, it would be preferable to test samples taken at **several** distances from the potential source of spray-drift, to find out if a gradient similar to the theoretical exponential decrease exists. In most cases, however, this would be too costly. Therefore, only two samples are normally taken: one close to the possible source of spray-drift, and one at the centre of the organic field. Exceptions are those cases, where an organic field is surrounded by several conventional fields.

A strict sampling protocol is followed (Supplementary Fig. 4a), establishing, among others, the number of sub-samples to be taken per plot (Supplementary Fig. 4b). Supplementary Fig. 5 describes the procedure to be followed in case of organic fields without risk of spray drift, and with one or more conventional neighbours, from whom spray drift could originate. In case the sample should get lost or damaged during transport, or the test result should be contested by the farm, two reference samples are kept, in addition to the main sample sent to the laboratory (Supplementary Fig. 4c). Samples are either kept cool in polyethylene bags, or dried in paper bags (Supplementary Fig. 4d). In either way, not only rotting of the samples is prevented, but also degradation of pesticide residues is slowed down or stopped entirely. In most cases, samples are shipped to the laboratory immediately after the inspection. When this is not possible, they are either frozen, or kept dry in dark and cool conditions, before transport. Detailed sampling records are kept (Supplementary Figs. 6, 7 and 8). Bags are sealed (Supplementary Fig. 9) and sent to accredited laboratories for multi-residue screening following DIN EN 15,662:2018–07 mod. LC–MS/MS, GC–MS/MS, GC-NCI-MS^38^. Depending on the matrix (type of material to be tested), this method covers within one single test approximately 700 different insecticides, fungicides and herbicides. A few samples were tested separately for dithiocarbamates (applicable method DIN EN 12,396–3:2000–10)^39^, or glyphosate (internal Eurofins method SPG-14.158.2 2019–05)^40^, because these pesticides are not detected by the multi-substance screening. To reduce testing costs, generally first a mixture of margin and centre samples is tested. Only in case of positive results, margin and centre samples are subsequently tested separately (Supplementary Fig. 10).

### Identification of variables for the interpretation of test results on aerial spray-drift in the banana industry in ecuador

Frequent aerial spraying in this industry leads to strong spray-drift, often with overlaps from several conventional neighbours on different sides of the organic farm. In addition, the high frequency of 17 to > 60 applications per year^41,42^ causes temporal overlaps of residues derived from several spraying events. To identify appropriate variables that allow discriminating between fraudulent application and spray-drift under these conditions (objective II), a total of 476 residue tests from Ecuador from 2018 and 2019 were analysed. As explained above, in most cases, first a mixed sample from borders and centre is tested (Supplementary Fig. 10). Based on the assumption that, due to the overall heavy drift problem, residues in mixed samples below 0.1 mg/kg were derived from drift and did not require separate testing, 119 mixed samples were identified as "drift" and no separate testing of margin and centre done. These were excluded from the statistical analysis. Residues in 20 mixed samples were so high that they were immediately identified as "application" and were also not considered, while 24 datasets were excluded because of sampling mistakes. This left datasets from 67 farms with 222 individual samples (i.e. 67 centre and 155 border samples), which were analysed separately and then subjected to statistical analyses. Of the 67 cases, 14 had been identified as "application", 48 as "drift", while five had remained "unclear".

Thirty-nine variables (Supplementary Table 7) were tested for their suitability for telling apart spray-drift from deliberate fungicide use by organic farmers. When the laboratory had found only "Traces < LOQ" for a specific substance, a default value of 0.005 mg/kg was used instead. For testing the variables, multivariate statistical analysis based on logistic regression, discriminant analysis and support vector machines were performed to find rules that would classify farms into the two groups^43^. Here, we only report results of the discriminant analysis, which provided the most satisfactory results. The variable selection and performance of models and their prediction accuracy were assessed using leave-one-out and *k*-fold cross validation. The selected variables were graphically displayed using a biplot^44^. A one-way ANOVA was used to test the statistical significance of these variables between the “application” and the “drift” farms. The analysis was performed in *R* programming language using MASS, caret and klaR libraries.

## Results and discussion

### Case Studies from Different Countries

An overview of the selected eight cases, the residues found in each sample, and the conclusion for each case, is provided in Table 1, followed by an explanation for each case study in sections Apple and blueberry orchards in Chile through Two examples from the banana industry.

#### Apple and blueberry orchards in Chile

The organic apple orchard from Chile borders with a conventional cherry plantation. While in the sample taken close to the cherries, 11 different pesticides were found, with different residues adding up to >13 mg/kg, at 100 m distance only seven substances were found, with a concentration of only 3% of the border sample—leaving no doubt that the residues were derived from drift. Yet, the values were so extremely high that the orchard lost its organic status under NOP, while under the Chilean organic standard, the farmer had to establish broad buffer zones. In a nearby blueberry plantation, however, we had the opposite picture: the concentration of imidacloprid in the margin sample was 0.15 mg/kg, while in the centre of the field, at 100 m from the margin, it was 1.8 mg/kg. This was a clear case of fraud, and the farm lost its certification.

#### Soy-beans from Togo

In the centre sample from farmer 1 (Supplementary Fig. 6), chlorpyrifos and traces of dichlobenil were detected. In the margin sample from the same farmer, however, two other substances were found, but no chlorpyrifos. Thus, most probably there was an overlap of an application (chlorpyrifos) and drift (for the other two substances). In the case of farmer 2, only traces of deltamethrin were found in the margin sample, but no residues in the centre, thus this was a clear "drift" situation. From farmer 3, only one sample was taken, because there was no conventional neighbour. The sample had relatively high residues of fipronil, clearly showing an application. These results demonstrate that even for small fields of less than 1 ha, the difference between residues derived from spray-drift and from application by the organic farmer can often be established, especially when neighbours use manual knapsack sprayers. As a result, the group's internal control system excluded several member farmers from the group and had to improve its internal member monitoring.

#### Rice field in Thailand

The insecticides bifenthrin and chlorpyrifos were identified at levels of 0.005 to 0.013 mg/kg in samples from centre and margin, respectively, of a 4 ha rice field (Supplementary Fig. 7). The on-site inspection did not reveal any evidence for use of these substances by the organic farmer. Short-range drift could be ruled out, because in this case the residues in the centre would be expected to be by a factor 10 lower than in the margin sample. Both insecticides have a low vapour pressure, therefore long-range drift through evaporation is also excluded. The residues could theoretically originate from an application two to three months prior to sampling, but also from long-range drift through dust, or contaminated irrigation water. Many conventional rice farmers in the region use these insecticides. Under the principle of "innocent until proven guilty", the farmer remains certified.

#### Cocoa plantation in Ecuador

Residues of 2,4-D had been detected in cocoa beans by the importer's CB in Belgium. This was the starting point for an investigation at farm level. 2,4-D is a specific herbicide for controlling dicotyledonous weeds in cereals. In Latin America, however, it is often used for controlling weeds in perennial crops. Weed samples from the centre of the plantation had low levels of the herbicide, while weed samples from the field margins and cocoa leaf samples were free of 2,4-D. Several fungicides found in the cocoa leaves probably came from aerial spraying on nearby banana plantations, but this could not have been the case for 2,4-D, because aerial spraying of this herbicide would kill the banana plants, while spray-drift from manual knapsack sprayers used in between banana plants, with the nozzle turned downwards, is almost zero. Also, long-range drift could be ruled out, because considering the dense canopy of cocoa trees, this would lead to higher residues in the canopy itself than in the weeds growing beneath. Dry weeds observed by the inspectors in between the cocoa trees provided further evidence of herbicide application on the organic plantation. Therefore, the certificate was suspended in spite of the low residue level.

#### Oil-bearing roses from Bulgaria

A leaf sample from an organic oil-bearing rose (*Rosa damascena*) field in Bulgaria in 2020 had penconazole residues of 0.62 mg/kg. The farmer's claim of the neighbour spraying at a wind speed of 11 to 13 m/s seemed unlikely (not only because spraying under such conditions is not effective, but also because data from regional weather stations show a maximum wind speed of 3.8 m/s for the entire month). Using a spray-drift equation for air-blast spraying^36^:

1 $$y = 3908x^{{ - 2.42}}$$

with: x = distance from the target field and y = deposit at distance x, expressed as a fraction of the deposit on the target field, combined with approximate data concerning the impact of wind speed^37^, CERES concluded that the assumption of these residues being derived from drift, was not plausible (Supplementary Table 8). Penconazole was also detected in a sample of rinse water from the organic farm's sprayer, further supporting the presumption that it was a case of deliberate application. The farm lost its organic status.

#### Vineyards in Germany

Grape leaves were sampled from eight organic vineyards during a period when conventional farmers were applying fungicides for preventing different fungus diseases. Samples from seven farmers had residues with a maximum of 0.75 mg/kg for folpet and 0.52 mg/kg for dithiocarbamates. The small size of the vineyards, combined with air-blast spraying by neighbours and possibly air swirling caused by thermal lift in the hilly landscape, did not allow for a clear distinction between margin and centre samples. On farm N°8, however, the folpet concentration reached 73 mg/kg, clearly indicating a direct application by the organic farmer. This was confirmed later by a sample taken from sprayer rinsing water. This farmer lost the organic status, while the others remained certified. This decision was correct assuming that under the given weather conditions, all farmers in the region had sprayed more or less at the same time, so that drift effects were not confounded with dissipation effects.

#### Walnuts from Moldova

Between 2017 and 2019, eight out of eight walnut samples from a company in Moldova dedicated to wild collection had low residues of the herbicide 2,4-D (average 0.016 mg/kg). Four hypotheses were considered regarding the origin of this phenomenon: (a) Ubiquity due to long-range transport: 2,4-D is known to be taken up by plant roots and transported via the xylem^45^. Because of its lipophilic condition^46^ it is often found in walnuts. This, together with consistently low residues in all samples from three harvest seasons, at a first glance made ubiquity in the region the most plausible explanation. (b) For facilitating harvest, collectors might remove vegetation below the walnut trees with the help of the herbicide: This could be ruled out, because it would have been easily visible during on-site visits. (c) Short-range spray-drift from nearby cereal fields: To verify this hypothesis, leaf samples were taken from margins and centres of the collection areas. Indeed, the two margin samples had traces of 2,4-D < LOQ, while the six centre samples were free of residues. However, the finding did not seem to be a plausible explanation for the presence of 2,4-D in **all** walnut samples from three seasons. (d) Collectors might be delivering nuts from non-certified areas: This was confirmed through collector interviews. Due to the pressure from the CB, the company implemented strict measures for preventing delivery of nuts from non-certified areas. As a result, nine out of nine nut samples from the 2020 harvest were free of residues, thus refuting the ubiquity hypothesis and showing that most probably (d) was the main cause of the problem, possibly in combination with (c). After implementing the necessary measures, the company kept its organic status.

#### Two examples from the banana industry

Sampling banana leaves is a time-consuming effort (Fig. 4d). On the first plantation, not only the sum of all pesticide residues was substantially higher in the centre than in the margins, but also the values for most individual substances (Fig. 4a). This did not leave any doubt that the residues were derived from an application by the organic farmer, whose certificate was then suspended. On the second plantation, however, only the sample taken close to the conventional banana neighbour had residues, while the samples from the centre and close to a plantain orchard were free of residues. The residues were derived from drift and the farm kept its organic status (Fig. 4b and Supplementary Fig. 8).

### Statistical analysis of banana sample test results from Ecuador

#### Results by CERES

For many cases, however, the decision between "drift" and application was not as clear as in Fig. 4a,b. As an example, a rather complex case is presented in Fig. 4c. In total of all samples, residues of 25 different fungicides were detected, with a group of nine substances (difenoconazole, epoxiconazole, fenpropidin, fenpropimorph, propiconazole, pyrimethanil, spiroxamine, tebuconazole, triadimenol) each occurring in more than one third of the 222 samples from the 67 farms. The highest value for one single substance was 4.8 mg/kg;

21% of the centre samples, but only 1.3% of the border samples were free of residues (Supplementary Table 9).

CERES had decided that of the 67 farms included in the analysis, on 14 farms fraudulent pesticide applications had taken place, while 48 were classified as drift, and five had remained "unclear".

#### Statistical approach

##### Development

In a first approach, the discriminant analysis identified six variables as the most promising ones based on a cross-validated stepwise selection procedure (1subcen, 2subrat2, 3maxcen, 4maxrat3, 5sumcen and 6sumrat2, see Supplementary Table 7). The one-way ANOVA also indicated that the six selected variables are significantly different between the drift and the application group (Supplementary Table 10).

**Figure 6:**
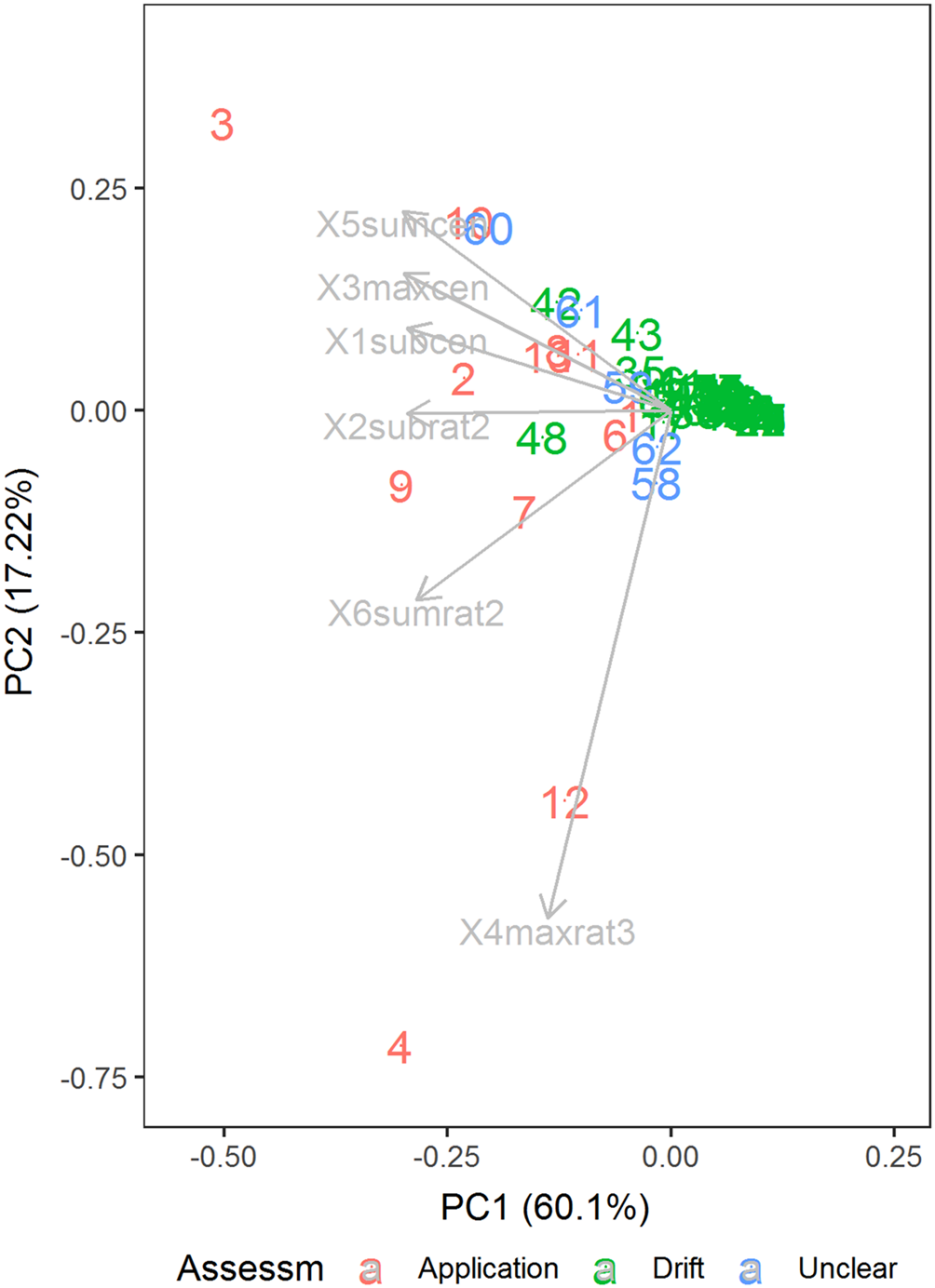
Principal Component Analysis (PCA) biplot of 67 farm samples for four variables used for the discriminant analysis (3maxcen, 2subrat2, 6sumrat2, 4maxrat3). The samples are coloured by the initial classification. The “drift” farms are clustered around (0, 0) while “application” farms are spread on left of the plot, and the "unclear" cases are distributed throughout.

Farms previously considered as having been subject to drift mostly clustered around zero while application farms scattered on the left side of plot with two exceptions clustering around zero. The farms considered unclear are distributed throughout. The raw data for the six variables were visualized using a heatmap (Supplementary Fig. 11). For this, each variable was standardized to a mean of zero and unit variance. The clustering of farms is visualized using a dendrogram based on the Unweighted Pair Group Method with Arithmetic means (UPGMA). The heatmap shows that “application” farms tend to be elevated in all six variables, confirming the one-way ANOVA results, even though there is non-negligible heterogeneity within groups. Application farms are clustered in the top rows, showing that two farms that had been considered subject to drift grouped clearly with the "applicants", whereas three supposed applicants grouped with the spray drift group. Four of the five unclear cases grouped in the application category or at the edge towards the drift group while one unclear case fell in the drift group. Taking a closer look at the initial raw data and sampling record for those farms, which visually in both heatmap and biplot appeared to be misclassified as "drift" (cases 42 and 48), revealed that these cases should not have been included in the analysis because the field samples had been taken in a wrong way, and that the cases 5, 8 and 14 should have been classified as “drift”. Thus, we subsequently re-classified the latter three cases as “drift” and the former two as “unclear”, leaving a training dataset containing the 60 farms for which the class had been assigned as either “drift” or “application”. The test dataset contains the five cases originally classified as “unclear” (cases 58, 59, 60, 61, 62) and the two cases subsequently removed from the training set (cases 42 & 48). The linear discriminant function performed best in terms of accuracy with the four variables 2subrat2, 3maxcen, 4maxrat3 and 6sumrat2 (Fig. 5 and Fig. 6). The biplot based on the first two principal components using these selected variables explains 60 and 22% of the total variation, respectively (Fig. 6).

**Figure 7:**
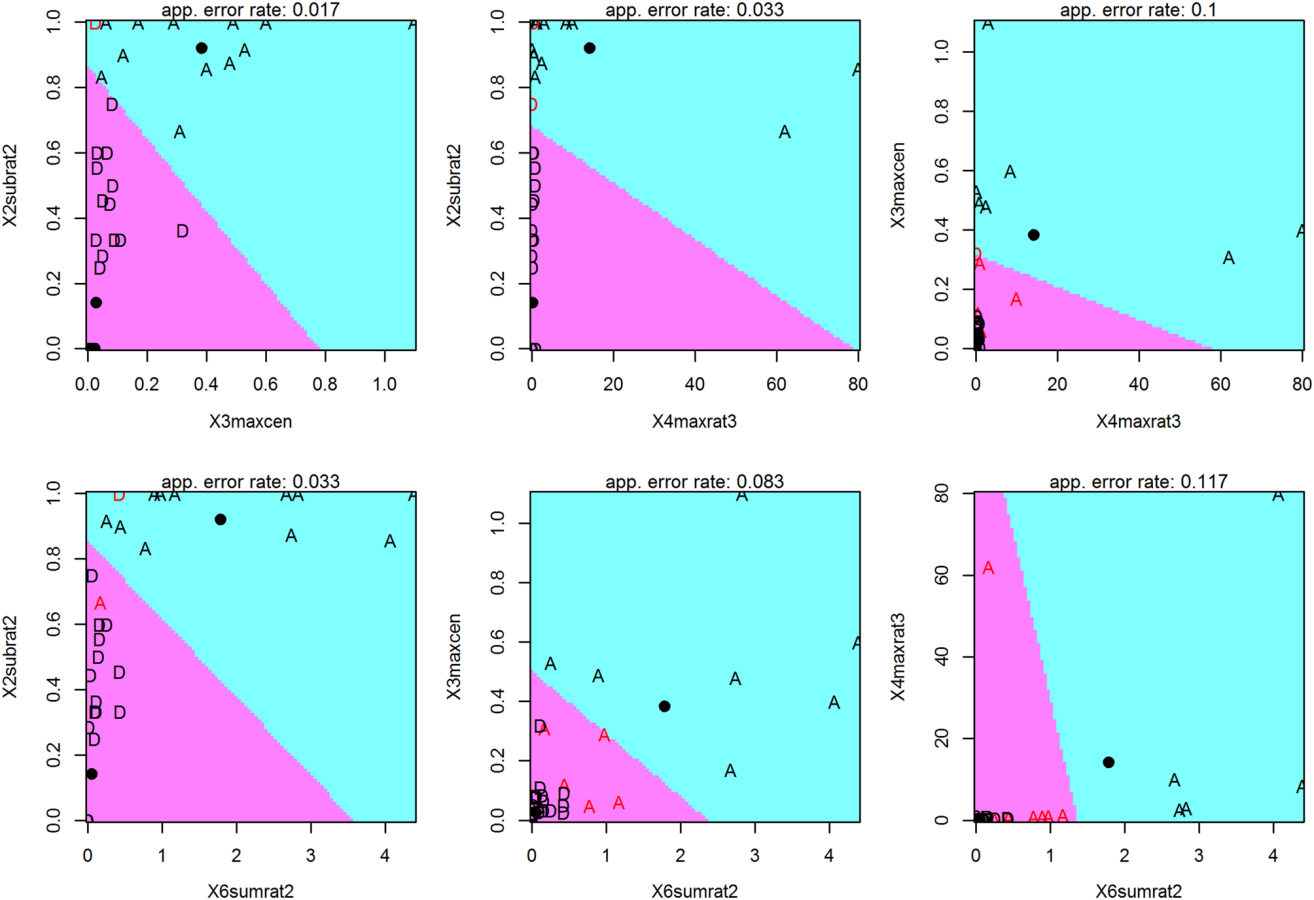
Linear discriminant function for the four selected variables. The D represents the true “drift” cases while A represents the true “application” cases. The two colours represent the decision rule: cases falling into the magenta region are classified as “drift”, cases falling into the turquoise region are classified as “application” cases. Misclassified farms are plotted in red, correctly classified ones in black. The black dots represent the group means.

The linear discriminant function was evaluated by cross validation and found to correctly classify a farm as either "drift" or "application" with an accuracy of 93.3%. The leave-one-out cross validation method was used to evaluate the accuracy of the model. In this method, each sample farm was dropped from the test data and then the class of that farm was predicted using the discriminant model. The misclassification rate in this cross validation of a “drift” as an “application” farm was 2.1%. This means that for a farm that is truly a “drift farm" there is an estimated probability of 2.1% that it is erroneously classified as an “application” farm. The misclassification rate of an “application” farm as a “drift” farm was estimated at 25% (Fig. 7). Thus, for an “application farm” there is an estimated 1 in 4 chance of being falsely classified as a “drift” farm. Of course, these estimated error rates are themselves subject to estimation error, and it is desirable to accumulate data from more farms to stabilize these estimates, as well as the estimates of the discriminant function. It also needs to be taken into account that, as we have explained here, there was some uncertainty regarding correct group membership for some farms that was only revealed by closer scrutiny of the initial statistical analysis. This may mean that the error rates we obtained in cross-validation of the final analysis presented here are on the optimistic side. The continuation of the present work, and especially the accumulation of data from more farms, will help to avoid such wrong assessments in the future. Three out of the five initially "unclear" cases turned out to belong to the "application" group, two to the "drift" group.

##### Final result

The linear discriminant function in our analysis is (See Fig. 5 and Supplementary Table 7 for explanation of variables):

2 $$Application = - 13.63421 + 16.17447\left( {2subrat2} \right) + 12.20420\left( {3maxcen} \right) + 0.11764(4maxrat3 + 1.5647\left( {6sumrat2} \right)$$

3 $$Drift = - 0.42757 + 2.76201\left( {2subrat2} \right) + 0.91776\left( {3maxcen} \right) + 0.00942\left( {4maxrat3} \right) - 0.16929\left( {6sumrat2} \right)$$

$$If\;Application\; > \;Drift\; \to \;Application$$

$$If\;Application\; < \;Drift\; \to \;Drift$$

The linear discriminant function is also depicted in Fig. 7 for the four selected variables. For each pair of variables, the plot shows the separation of the two groups by two different colours, and the placement of individual samples represents the rate of correct classification.

## Conclusions

### What we Found Out

- For the purpose of finding the origin of pesticide residues in organic products, field samples have several advantages, as compared to final product samples.
- In most cases, comparing pesticide residues in leaf samples from field margins close to a possible source of spray-drift, to samples from the centre of the organic field, allows to distinguish the effects of spray-drift from deliberate pesticide use by the organic farm. The method works even in regions with extremely intensive pesticide use and aerial spraying by conventional neighbours.
- The distinction is also possible when it comes to very small fields, where the distance between border and centre is short—provided that manual knapsack (as is normally the case in smallholder setups) or tractor boom sprayers are used. It becomes difficult to impossible on such small fields, when neighbours use air-blast or aerial spraying.
- When residues below approximately 0.03 mg/kg are found evenly spread over the field, it becomes difficult to distinguish long-range drift (from evaporation or wind erosion) from the results of deliberate use several weeks before sampling. In such cases, the test results alone do not allow to prove fraudulent practices, as long as other evidence (pesticide containers in the farm house, residues in rinse water in the sprayer, records, samples from non-cultivated areas, etc.) do not exist.
- When a reference sample from the field margin is not available, and residues are high in the central part of the organic farm, comparing the test results to expected values from standard deposition curves, can be enough to distinguish drift from application.
- For the specific conditions of fungicide spraying in the banana industry, where a high spraying frequency, heavy drift because of aircraft spraying, and drift from more than one conventional neighbours sometimes create a confusing picture, the variables explained in Fig. 5 and a linear discriminant function such as the one outlined above yield good results for differentiating drift from application.

### Way Forward

- Following strict sampling protocols and keeping detailed records are the key for using this method in a meaningful way. Sampling must be planned in a way that allows for clear interpretation of results. Taking only one sample from a field, often leads to useless results. Sampling residues in spraying equipment, sampling natural vegetation next to the organic field, cross-checking with book-keeping records and other inspection methods, should be used as complementary methods.
- The sampling mistakes leading to the exclusion of several test results from the statistical analysis, refer mostly to not taking the centre samples at sufficient distance from the edges. This has meanwhile been corrected through improved work instructions (Supplementary Fig. 12). Another correction of the procedure, which is currently being tested, is reduction of the "action level" for separate testing of centre and border samples from 0.1 to 0.06 mg/kg. Once a substantial number of test results under this new protocol have been obtained, other variables from Supplementary Table 7 might perform better, e.g. N° 7 through 15.
- We assume that the statistical approach described for the banana industry, can also be used for other crops exposed to heavy drift pressure (e.g. fruit orchards and vineyards), but this is yet to be confirmed.

## Electronic supplementary material

Below is the link to the electronic supplementary material.

Supplementary Information 1.

Supplementary Information 2.

Supplementary Information 3.

Supplementary Information 4.

Supplementary Information 5.
