# Author Correction: Appropriate sampling methods and statistics can tell apart fraud from pesticide drift in organic farming

**DOI:** 10.1038/s41598-021-97273-9

**Published:** 2021-09-01

**Authors:** Albrecht Benzing, Hans-Peter Piepho, Waqas Ahmed Malik, Maria R. Finckh, Manuel Mittelhammer, Dominic Strempel, Johannes Jaschik, Jochen Neuendorff, Liliana Guamán, José Mancheno, Luis Melo, Omar Pavón, Roberto Cangahuamín, Juan-Carlos Ullauri

**Affiliations:** 1 CERES GmbH, Vorderhaslach 1, 91230 Happurg, Germany; 2 Biostatistics Unit, Institute of Crop Science, University of Hohenheim, 70593 Stuttgart, Germany; 3 Department of Ecological Crop Protection, University of Kassel, Nordbahnhofstr. 1a, 37213 Witzenhausen, Germany; 4 Eurofins Dr. Specht International GmbH, Am Neulaender Gewerbepark 2, 21079 Hamburg, Germany; 5 Gesellschaft für Ressourcenschutz (GfRS), Prinzenstr. 4, 37073 Göttingen, Germany

Correction to: *Scientific Reports* https://doi.org/10.1038/s41598-021-93624-8, published online 20 July 2021

The original version of this Article contained errors.

A previous rendition of Figure 6 was published. The original Figure 6 and accompanying legend appear below.

**Figure 6:**
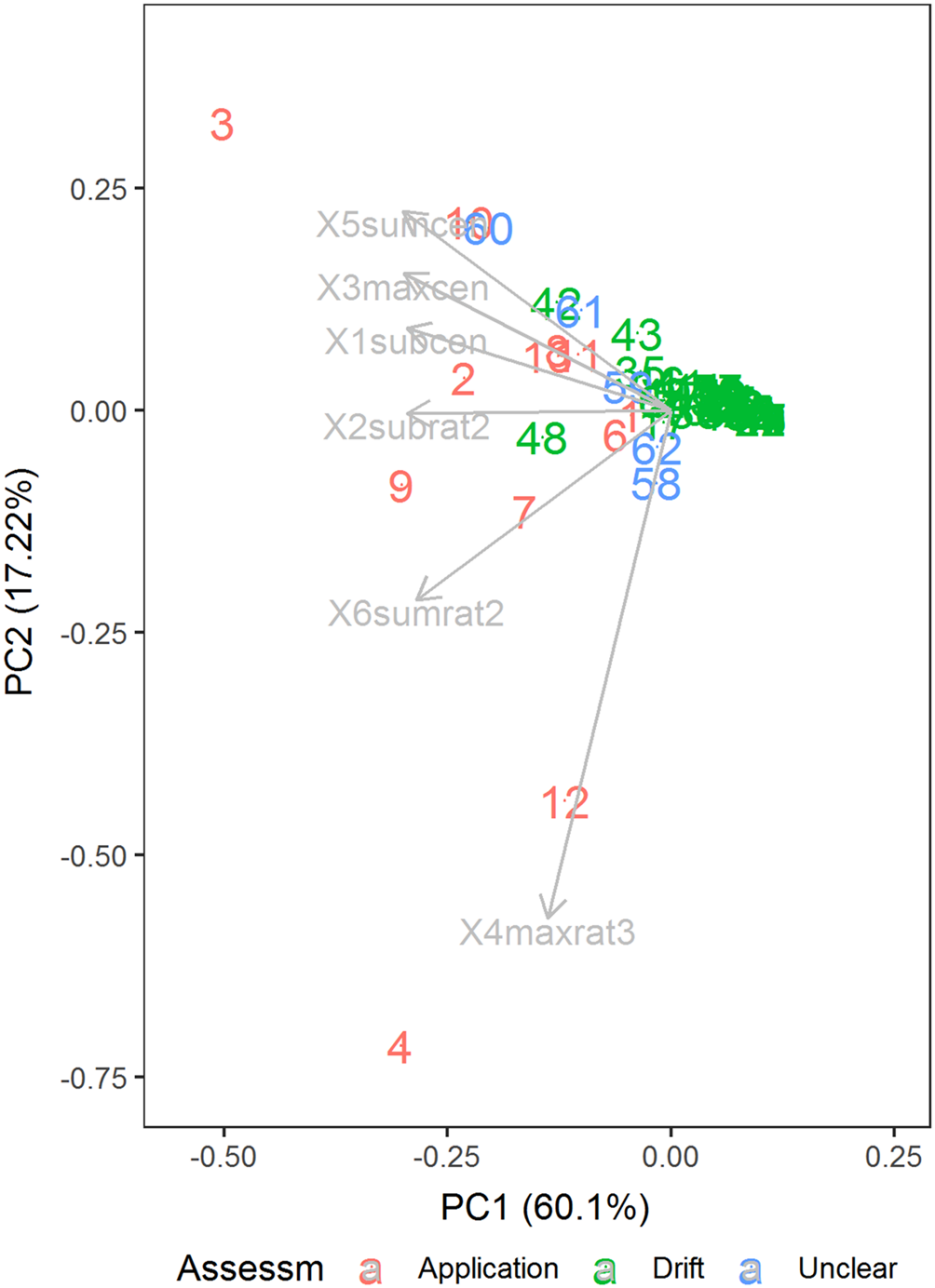
Principal Component Analysis (PCA) biplot of 67 farm samples for four variables used for the discriminant analysis (3maxcen, 2subrat2, 6sumrat2, 4maxrat3). The samples are coloured by the initial classification. The “drift” farms are clustered around (0, 0) while “application” farms are spread on left of the plot, and the “unclear” cases are distributed throughout.

In Supplementary Information 5, in the legend of Table 3,

*“Comparability of the datasets from two laboratories used in Figure 1c for pesticide residues: (mostly)* ***before*** *release to the organic market (Eurofins) and* ***on*** *the (retail and wholesale) organic market (CVUA).”*

now reads:

*“Comparability of the datasets from two laboratories used in Figure 1d for pesticide residues: (mostly)* ***before*** *release to the organic market (Eurofins) and* ***on*** *the (retail and wholesale) organic market (CVUA).”*

The original Supplementary Information 5 file is provided below.

The original Article and accompanying Supplementary Information file have been corrected.

## Supplementary Information

Supplementary Information 5.

