# Author Correction: Appropriate sampling methods and statistics can tell apart fraud from pesticide drift in organic farming

**DOI:** 10.1038/s41598-022-15100-1

**Published:** 2022-06-27

**Authors:** Albrecht Benzing, Hans-Peter Piepho, Waqas Ahmed Malik, Maria R. Finckh, Manuel Mittelhammer, Dominic Strempel, Johannes Jaschik, Jochen Neuendorff, Liliana Guamán, José Mancheno, Luis Melo, Omar Pavón, Roberto Cangahuamín, Juan-Carlos Ullauri

**Affiliations:** 1 CERES GmbH, Vorderhaslach 1, 91230 Happurg, Germany; 2 Biostatistics Unit, Institute of Crop Science, University of Hohenheim, 70593 Stuttgart, Germany; 3 Department of Ecological Crop Protection, University of Kassel, Nordbahnhofstr. 1a, 37213 Witzenhausen, Germany; 4 Eurofins Dr. Specht International GmbH, Am Neulaender Gewerbepark 2, 21079 Hamburg, Germany; 5 Gesellschaft für Ressourcenschutz (GfRS), Prinzenstr. 4, 37073 Göttingen, Germany

Correction to: *Scientific Reports* https://doi.org/10.1038/s41598-021-93624-8, published online 20 July 2021

The original version of this Article contained an error in Fig. 1, where data for the level of residues, the corresponding ratios between conventional and organic, and the number of samples from Eurofins reflected in panel (d), were incorrect.

The legend for Fig. 1d now reads: “Multi-layer sieving model for residue testing of fruits and vegetables in 2019, at different points of the organic supply chain. The data above the white arrows are from the commercial laboratory Eurofins, and mostly represent the situation **before** products are released to the market, while the figures below the white arrows are from CVUA, representing the situation **on** the market (both wholesale and retail). Ratios from “before market” to “on market” are shown in the white arrows. In this process, the MCPL remains in the same range for conventional products (blue rectangle to the right), while it is reduced massively for organic products (green trapezium in the centre). As a result of this sieving mechanism, residues in samples from the market are 150 and more times lower in organic than in conventional produce (trapezium at the bottom). This shows that the process represented by the blue arrows works fairly well—which is not always the case for the investigation of the origin of such residues, symbolised by the yellow arrows.”

**Figure 1:**
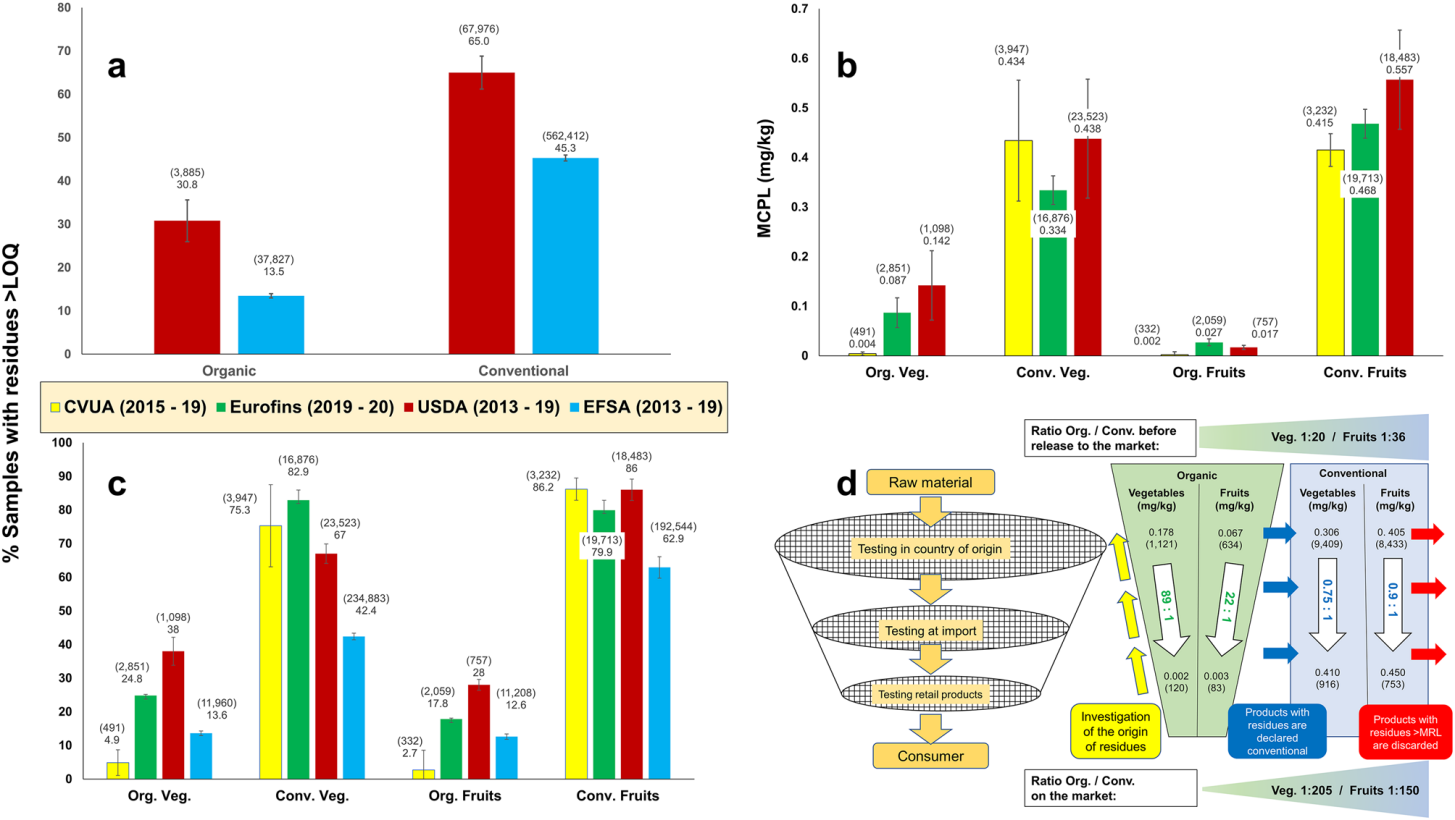
Pesticide residues in conventional and organic food in tests conducted by four organisations: EFSA (European Food Safety Authority) collects official data from all EU member states^3^, CVUA from one federal state in Germany^4^, USDA from government laboratories across the U.S.^5^, while Eurofins is a commercial laboratory in Germany. Figures in brackets represent the number of samples. The legend is valid for (**a**), (**b**) and (**c**). In order to increase the number of samples (represented in brackets) and thus their representativeness, figures from several years were grouped together, as available from each organisation. Black bars symbolise standard errors across years. (**a**) Shows the percentage of samples with residues above the limit of quantification (LOQ), for all types of food (available from two organisations only). (**b**) Represents the mean cumulative pesticide load (MCPL) for fruits and vegetables (available from three organisations). (**c**) Similar to (**a**), but for fresh fruits and vegetables only (CVUA uses “above 0.01 mg/kg” instead of LOQ, but this is identical for most substances). The same datasets were used for (**b**) and (**c**). (**d**) Multi-layer sieving model for residue testing of fruits and vegetables in 2019, at different points of the organic supply chain. The data above the white arrows are from the commercial laboratory Eurofins, and mostly represent the situation **before** products are released to the market, while the figures below the white arrows are from CVUA, representing the situation **on** the market (both wholesale and retail). Ratios from "before market" to "on market" are shown in the white arrows. In this process, the MCPL remains in the same range for conventional products (blue rectangle to the right), while it is reduced massively for organic products (green trapezium in the centre). As a result of this sieving mechanism, residues in samples from the market are 150 and more times lower in organic than in conventional produce (trapezium at the bottom). This shows that the process represented by the blue arrows works fairly well—which is not always the case for the investigation of the origin of such residues, symbolised by the yellow arrows.

The original Fig. 1 and accompanying legend appear below.

Furthermore, in the Introduction, under the subheading ‘Organic businesses' testing strategies’,

“Residues in organic produce reported from the market were reduced by 22 and 89 times in fruits and vegetables, respectively, compared to the levels reported by the commercial laboratory, which represent mostly pre-market samples, while the values for conventional samples remained in the same range.”

now reads:

“Residues in organic produce reported from the market were reduced by 6 and 23 times in fruits and vegetables, respectively, compared to the levels reported by the commercial laboratory, which represent mostly pre-market samples, while the values for conventional samples remained in the same range.”

The original Article has been corrected.

