# Supplementary material for: Appropriate sampling methods and statistics can tell apart fraud from pesticide drift in organic farming
Source: Sci Rep. 2021 Jul 20;11:14776. doi: 10.1038/s41598-021-93624-8 (PMC8292382; doi:10.1038/s41598-021-93624-8)
Supplement: Supplementary file 5 — Supplementary Information 5. [file 41598_2021_93624_MOESM5_ESM.pdf]

To safeguard confidentiality, names and words, which would allow identifying specific persons or companies, have been hidden or removed from the following figures.

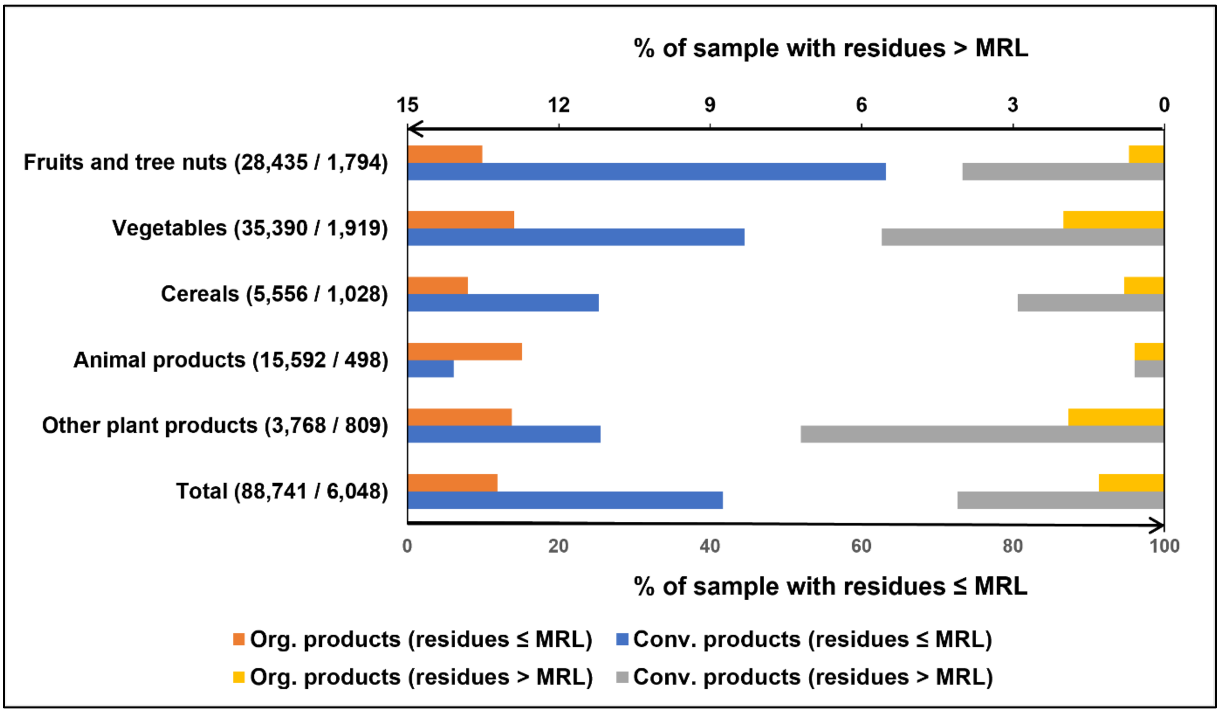

Supplementary Fig. 1: Pesticide residues in conventional and organic food tested by the national food authorities in 28 EU member countries in 2019 - totals and by groups of food. Modified from EFSA 2021 under the Creative Commons Attribution License. Please note the scale for "% of samples ≤MRL" (maximum residue limit) is on the lower side of the graph, from left to right, while the scale for "% of samples >MRL" is on the upper side, from right to left. Figures in brackets represent number of samples (conventional / organic). Pesticide residues in animal products mostly belong to the group of highly persistent organic pollutants (POPs: DDT, lindane, aldrin, etc.) and are derived from legacy problems, due to long half-lives in soil. Because of their lipophilic condition, they are more frequently found in high-fat products such as meat and milk. Especially in Eastern Europe, DDT and other pesticides of this group were used until the 1980s, and are therefore still frequently found in soil and food samples. In this (and several other) studies, residues of this type are more prominent in organic than in conventional food of animal origin. Reasons for this may be: (a) Access to outdoor areas is compulsory for organic livestock, therefore organic animals take up such substances directly with soil. (b) Because of lower nitrogen availability in organic farming systems, combined with restrictions concerning feeding rations, organic animal products tend to be richer in fat and less rich in protein. Due to accumulation of POPs in fat, they may appear more frequently in organic meat, milk and eggs.

a

[redacted] 88% 4:04 p. m.

Einkauf
para mí [redacted]
Ocultar detalles

De: Einkauf einkauf@[redacted]
Para: [redacted] @gmail.com
Cc: [redacted]
Einkauf [redacted]
Fecha: 28 jun 2019 8:05 a. m.

Estimada [redacted]

Se adjunta el pedido de compra de Lote R15160.

Pero NO embarque los tambores **62,63,64 porque los análisis demostraron altos valores de Streptomycina**.

b

Sehr geehrte Herren,

ich hoffe es geht Ihnen gut und Sie sind wie ich gut erholt aus den Sommerferien zurück ☺

Ich fliege in wenigen Tagen nach [redacted] um unseren Lieferanten [redacted] zu besuchen. Vor wenigen Tagen wollten wir importieren nachdem unsere Beprobung nach EU 401 abgeschlossen war. Wir haben laufende Kontrakte zu erfüllen die wir mit [redacted] und unseren Kunden abgeschlossen haben.

[redacted] teilte uns mit, dass CERES kein COI mehr ausstellt. Für diese Lieferung? Generell? Das verstehen wir nicht und es bringt unsere gesamten Planung und unsere Verpflichtungen Kunden gegenüber durcheinander. Wir beliefern namhafte Unternehmen aus der deutschen Bioindustrie. Unsere Kunden fragen uns jetzt warum wir bisher liefern durften und warum plötzlich nicht mehr und was es mit vergangenen Lieferungen auf sich hat...Bio Status etc. Die Verunsicherung bei usn und unseren Kunden ist groß und wir brauchen bitte schnelle Antworten.

Bitte teilen Sie uns doch möglichst morgen mit, ob Sie die Ware mit den Analysen im Anhang frei geben. Gerne telefonieren wir auch miteinander. Frau [redacted] setze ich cc. Sie leitet unsere QS.

Vielen Dank und herzliche Grüße

Ihr

c

An organic export company from Egypt is certified by a different CB. The German importer wanted to buy organic sesame from this company. Since he did not trust the other CB, he hired CERES for sampling the sesame before buying it. CERES took the samples, but wrote in the sampling report, that the company's documentation did not allow to trace the batch back to an organic source. The samples were free of residues, therefore the importer decided to buy the sesame – in spite of the missing traceability.

Supplementary Fig. 2: Anecdotic evidence of organic businesses' testing strategies: ***(a)*** *Screenshot of an email, sent by a Latin American whistle-blower to CERES. In this email, a German importer writes to his Latin American organic honey supplier: "We attach buying order for batch R15160. But please do NOT load barrels number 62, 63, 64, because testing showed high streptomycin values." Streptomycin is an antibiotic used by conventional beekeepers in some countries, but not allowed in organic beekeeping. The presence of "high values" cannot be explained by accidental contamination. Either one of the organic beekeepers had used the antibiotic, or the honey had been bought from conventional sources. Neither the importer's nor the exporter's CB had been informed about this and other similar findings; it was only because of the whistle-blower that the incidents were detected and investigated.* ***(b)*** *CERES had found systematic fraud in a Central Asian organic export company. The*

company had authorised the CB to share the information about the fraud with a German importer. In this email, the importer (knowing the product was not organic) insists the product should be released for import as organic, because test results show it is free of residues. (c) A case from Egypt, showing that "free of residues" is often considered sufficient for buying products as "organic". According to the rules, a batch that is not traceable back to an organic source, would have to be considered conventional.

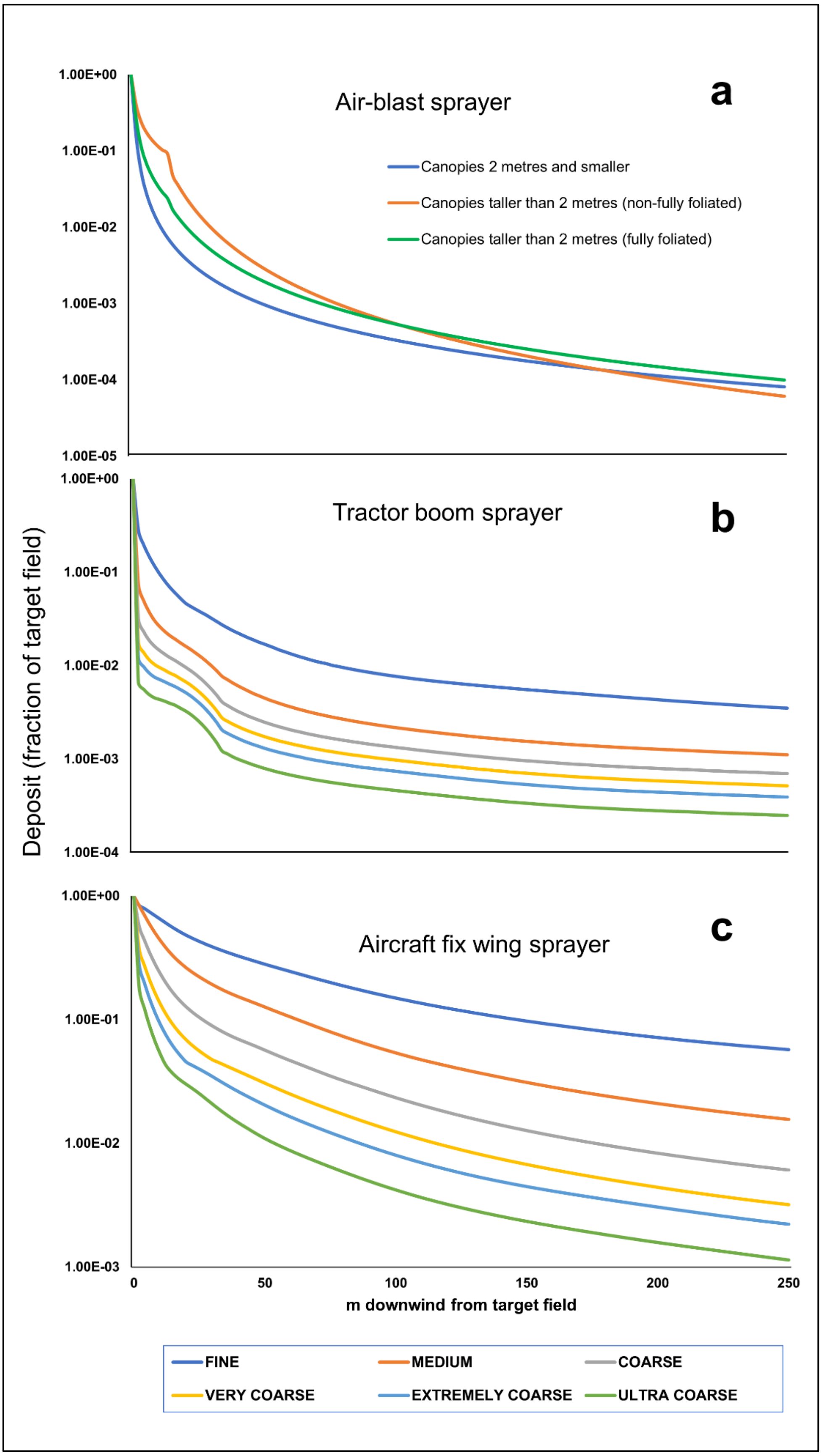

Supplementary Fig. 3: Standard deposition curves for different types of spraying equipment, without considering wind speed. Data from APVMA 2019.[37] Note the logarithmic scale of the vertical axis, which is different for each equipment. For air-blast sprayers, droplets are always fine, while for the other two sprayers, drift largely depends on droplet size. The curves do not run smoothly at certain points because the equations used by APVMA change at these points.

a

a. A representative of the farm/company must be present during the whole process of sampling.

b. Before sampling, make an overview of the lots, fields (respectively field parts) to be sampled, of the number of samples to be taken and arrange any additional tools that you need for the sampling (e.g. enough one-way gloves, bucket, sampling bags, shovel, etc.). Make sure that all tools and buckets are very thoroughly cleaned, before using them.

c. Except for sampling of packed products, new one-way gloves must be worn for sampling. They must be changed after each sample (e.g. after sampling lot A, change the gloves and proceed to lot B; after sampling the centre of a field, change the gloves and proceed to the buffer zone.) If no gloves are available, make sure to thoroughly wash your hands with soap after each sample. Whenever samples are to be tested for Dithiocarbamates, avoid using latex gloves because this may lead to false positive results!

d. Collect approx. 1 kg of material from each field respectively batch/lot or field part (buffer zone, margin, centre of a field). Make sure that this sample is mixed homogeneously. Then separate the sample in three portions of similar size:

- One to be sent to the laboratory
- One to be kept by the operator
- One to be kept by CERES.

b

Take subsamples in a randomised scheme from all over the field, per the following table:

Number of subsamples according to the plot size:

Plot size	Number of subsamples per plot
< 5 ha	10
5 – 20 ha	20
> 20 ha	30

c

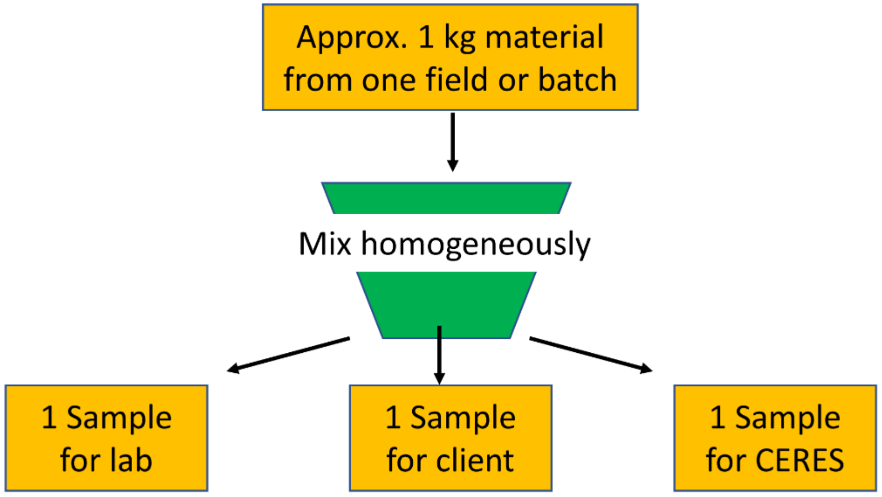

d

a. Samples must be stored in a safe place, without access of unauthorised persons

b. Make sure there is no risk of labels or identifications being removed or deleted or otherwise becoming illegible

c. Samples in PE bags should be kept cool whenever possible (refrigerator in the hotel room or office).

d. Please do not put samples in paper bags into the fridge! They should be kept at room temperature and preferably at dry places.

e. If samples are stored at room temperature (e.g. leaves in paper bags, or grains), make sure they are kept in a dry place that does not become too warm; make sure the room is not exposed to contamination, especially from household insecticides or the like!

f. Fruit samples, or fresh leaf samples in PE bags must be frozen, if they are to be stored for more than a few days.

g. In case you store several samples in paper bags together, please try to avoid them from getting in direct contact with each other. Although this is very unlikely and only relevant in case of highly contaminated samples, pesticides might be transported from one sample to another through the paper.

Supplementary Fig. 4: Extracts from the internal CERES work instruction for sampling: ***(a)*** *general instructions,* ***(b)*** *number of subsamples based on field size,* ***(c)*** *splitting the main sample for the laboratory, and the reference samples for CERES and for the farmer, and* ***(d)*** *storage of samples.*

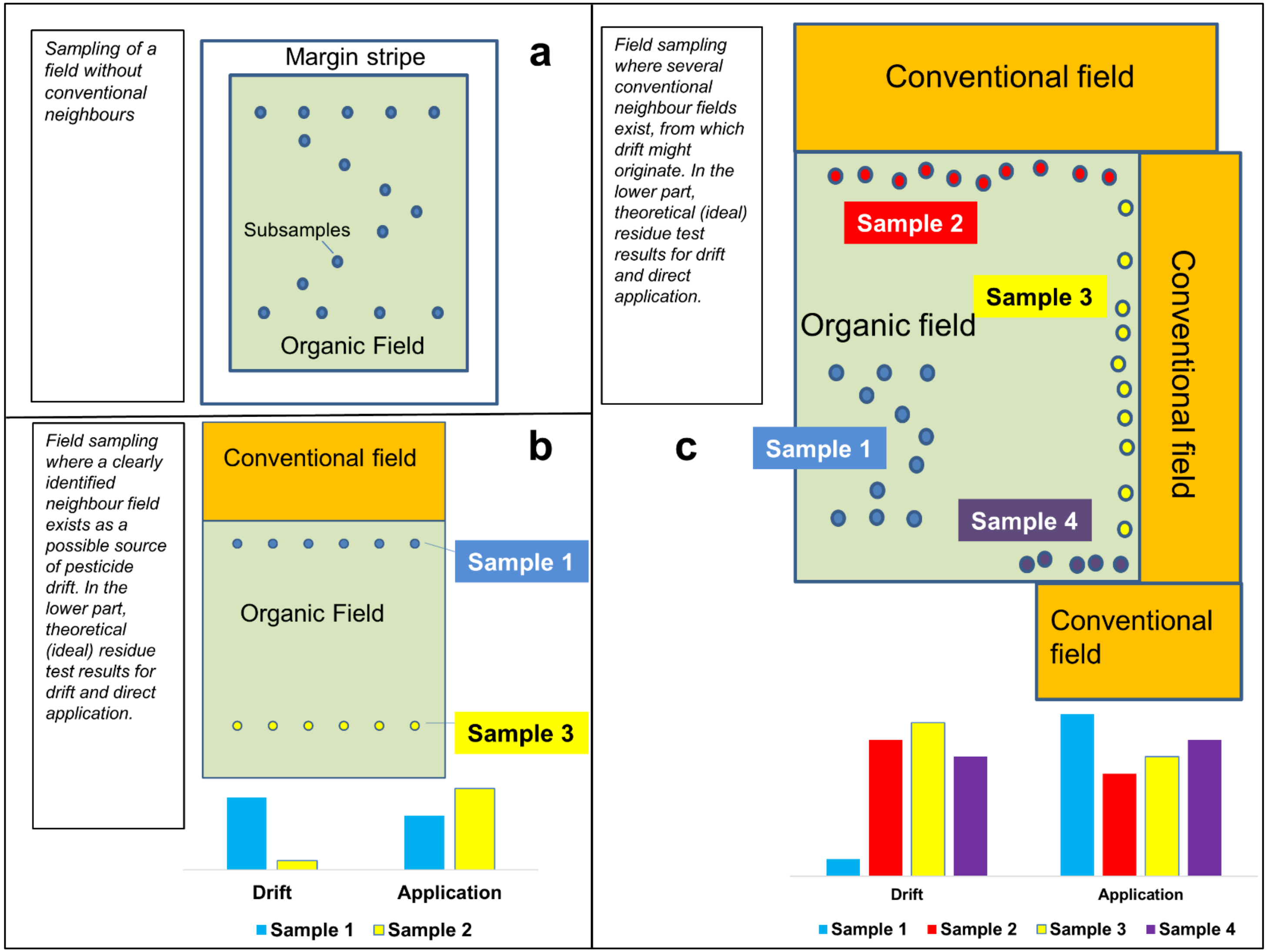

Supplementary Fig. 5: Extract from the internal CERES work instruction for sample taking under three different setups: (a) No nearby source of spray-drift, (b) one conventional neighbour as a possible source of spray-drift, and (c) three different conventional neighbours.

CERES	4.10.3.1en T	Sampling Record Field	01.04.2014	1/3

Sampling Record Field

1	Date: 12. – 13.11.2018	Farm or company name: [redacted]
2	Responsible person present during sampling: Internal inspectors [redacted]	CERES staff who took sample: Dominic Strempel
3	Type of samples: Soybean whole plants, and some weeds	Samples were taken from: Field ☒ Other:
4	Describe details of sampling method: Took samples according to CERES WI 4.10.1; For both with drift risk farmers, I sampled field margin and center separately to verify possible drift impact	

5	Sample (bag) number	Taken from (field number or name)	Crop	Composed of … subsamples	Is there a drift risk on this field (yes/no)? If yes, please provide further details in Section 6
	19781395 (Eurofins) 19781401 [redacted]	[redacted]	Soybean plants and weeds (center of field)	15	Yes, there is a conventional blackeyed peas field (insecticides, herbicides) to the southwest of the plot
	19781418 (Eurofins) 19781425 [redacted]		Soybean plants and weeds (buffer zone)	8	
	19782224 (Eurofins) 19782231 [redacted]		Soybean plants and weeds (center of field)	15	Yes, there is a conventional blackeyed pea field (insecticides, herbicides) and a conventional soybean field (herbicide??) next to the organic field
	19782255 (Eurofins) 19782248 [redacted]		Soybean plants and weeds (buffer zone)	6	
	19782071 (Eurofins) 19782064 [redacted]		Soybean plants	8	No, but suspicion of glyphosate use

6 *(This section is to be completed **only** if there is a risk of pesticide drift)*

Conventional neighbour **field** (name or number)	**Crop** on neighbour field	1st applic. on this field: **Date**	**Products** applied	2nd applic. on this field: **Date**	**Products** applied	Applic. equipment	Risk (inspector's opinion)	Source of information	Reliability of this information
No info, neighbour of [redacted]6	Blackeyed peas	May-June	Glyphosate	September	Lambda-Cyhalothrin	Backpack sprayer	medium	farmer	ok

CERES	4.10.3.1en T	Sampling Record Field	01.04.2014	2/3

No info, neighbours of [redacted]5	Blackeyed peas	May-June	Glyphosate	September	Lambda-Cyhalothrin	Backpack sprayer	medium	farmer	ok
	soybeans	Feb- Mar	Glyphosate			Backpack sprayer	medium	farmer	ok
	Cotton (80m)	From Mar-September	Glyphosate, Lambda-Cyhalothrin			Backpacks prayer	low	farmer	ok

7	Samples were taken based on a specific suspicion: Yes ☒ No ☐ If "yes", which could be the substance to be tested for? Lambda Cyhalothrin, Glyphosate
8	Number of **samples taken along by CERES representative**: 4 Sealed ☒ Unsealed ☐ Sample Bag Number(s): 19781395, 19781418, 19782224, 19782255<br>Number of counter samples **kept by company**: 4 Sealed ☒ Unsealed ☐ Sample Bag Number(s): 19781401, 19781425, 19782231, 19782248
9	Further comments: Found a bottle of Glyphosate in the conventional soybean field next to [redacted]5

(Please always provide a drawing on page 2!)

[redacted]

Signature Operator

Signature CERES

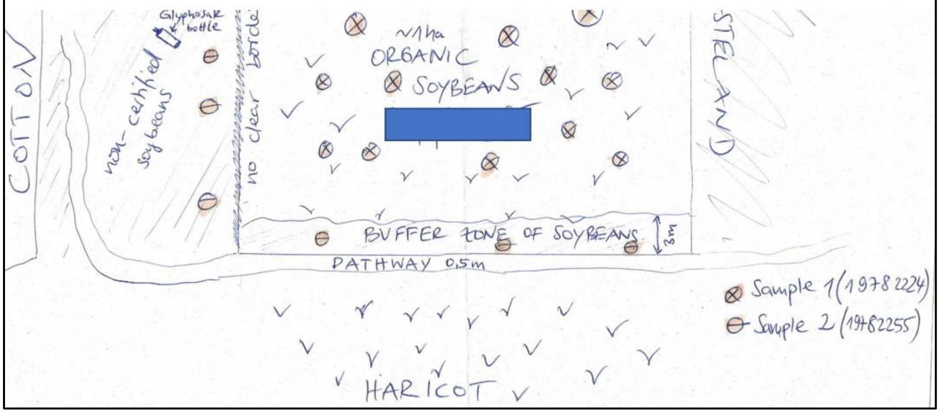

Supplementary Fig. 6: Sampling record and corresponding sampling map for soybean samples from a 0.5 ha soybean field in Togo. Borders, distances, drift risks, possible spraying times, and substances used by conventional neighbours are identified in the record and on the map. Drawing by D. Strempel.

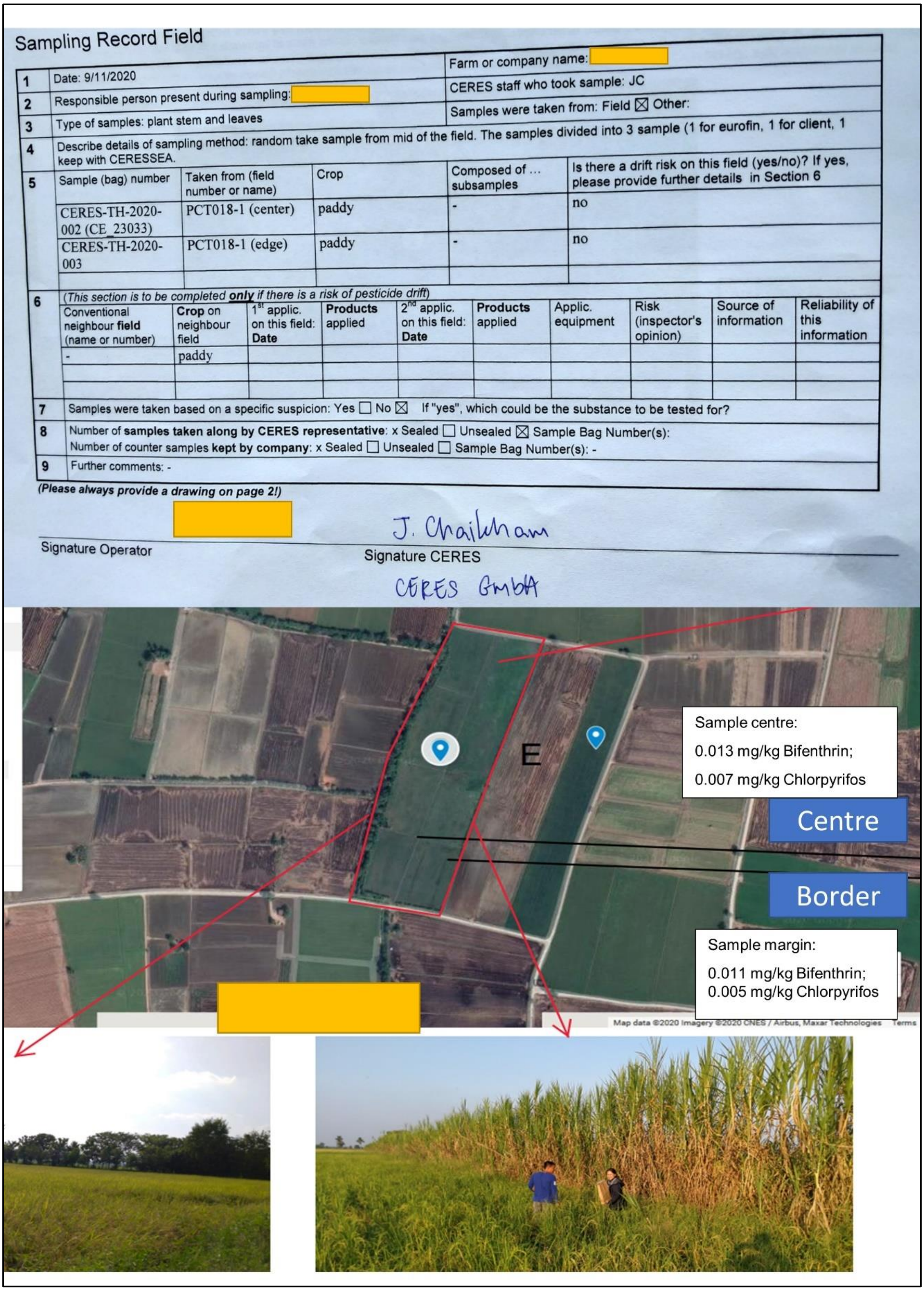

Sampling Record Field

1	Date: 9/11/2020	Farm or company name: [redacted]
2	Responsible person present during sampling: [redacted]	CERES staff who took sample: JC
3	Type of samples: plant stem and leaves	Samples were taken from: Field ☒ Other:
4	Describe details of sampling method: random take sample from mid of the field. The samples divided into 3 sample (1 for eurofin, 1 for client, 1 keep with CERESSEA.	

5	Sample (bag) number	Taken from (field number or name)	Crop	Composed of … subsamples	Is there a drift risk on this field (yes/no)? If yes, please provide further details in Section 6
	CERES-TH-2020-002 (CE_23033)	PCT018-1 (center)	paddy	-	no
	CERES-TH-2020-003	PCT018-1 (edge)	paddy	-	no

6 (*This section is to be completed **only** if there is a risk of pesticide drift*)

Conventional neighbour **field** (name or number)	**Crop** on neighbour field	1st applic. on this field: **Date**	**Products** applied	2nd applic. on this field: **Date**	**Products** applied	Applic. equipment	Risk (inspector's opinion)	Source of information	Reliability of this information
-	paddy								

7 Samples were taken based on a specific suspicion: Yes ☐ No ☒ If "yes", which could be the substance to be tested for?

8 Number of **samples taken along by CERES representative**: x Sealed ☐ Unsealed ☒ Sample Bag Number(s):
Number of counter samples **kept by company**: x Sealed ☐ Unsealed ☐ Sample Bag Number(s): -

9 Further comments: -

(Please always provide a drawing on page 2!)

[redacted]

Signature Operator

J. Chaikham

Signature CERES

CERES GmbH

Supplementary Fig. 7: Sample record and map for a 4 ha rice field in Thailand. Traces of the insecticides bifenthrin and chlorpyrifos were found at similar levels in the border and in the centre sample (distance approx. 50 m). The field has a Napier grass barrier to the East, and a tree buffer stripe to the West. Map created using Google My Maps, version APK 2.2.1.4, https://play.google.com/store/apps/details?id=com.google.android.apps.m4b&hl=en. Photos by J. Chaikham, CERES Thailand.

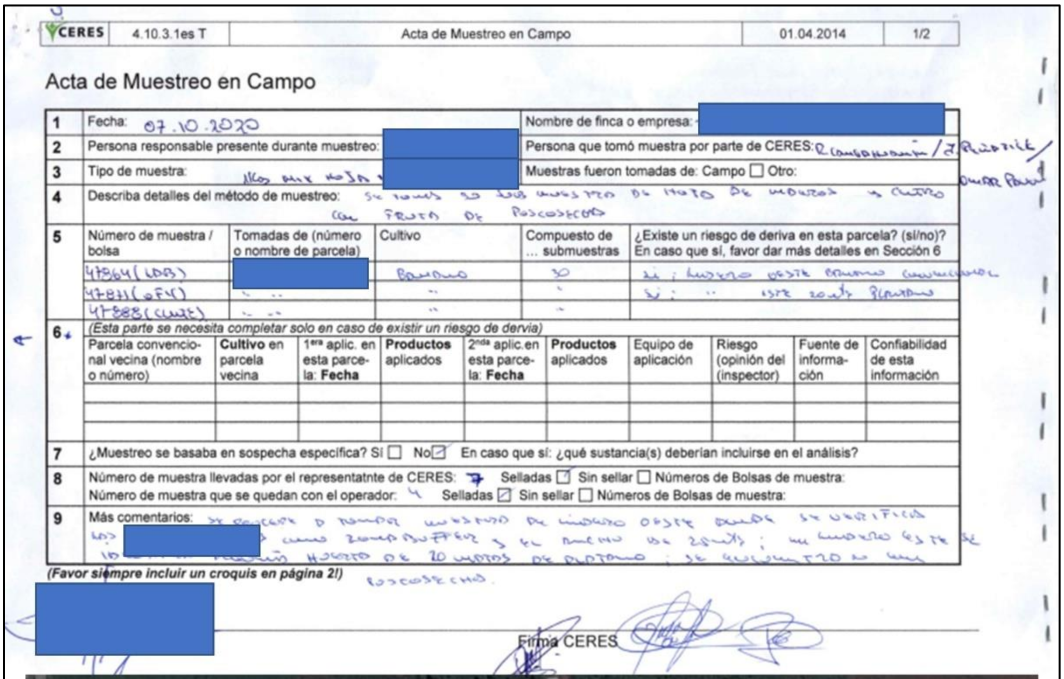

CERES	4.10.3.1es T	Acta de Muestreo en Campo	01.04.2014	1/2

Acta de Muestreo en Campo

1	Fecha: 07.10.2020	Nombre de finca o empresa: [redacted]
2	Persona responsable presente durante muestreo: [redacted]	Persona que tomó muestra por parte de CERES: [illegible]
3	Tipo de muestra: [illegible] [redacted]	Muestras fueron tomadas de: Campo ☐ Otro:
4	Describa detalles del método de muestreo: [illegible]	

5	Número de muestra / bolsa	Tomadas de (número o nombre de parcela)	Cultivo	Compuesto de … submuestras	¿Existe un riesgo de deriva en esta parcela? (sí/no)? En caso que sí, favor dar más detalles en Sección 6
	47864 ([illegible])	[redacted]	Banano	30	Si; [illegible]
	47871 ([illegible])	" "	"	"	Si; [illegible]
	47888 ([illegible])	" "	"	"	

6 (Esta parte se necesita completar solo en caso de existir un riesgo de dervia)

Parcela convencional vecina (nombre o número)	**Cultivo** en parcela vecina	1era aplic. en esta parcela: **Fecha**	**Productos** aplicados	2nda aplic. en esta parcela: **Fecha**	**Productos** aplicados	Equipo de aplicación	Riesgo (opinión del (inspector)	Fuente de información	Confiabilidad de esta información

7 ¿Muestreo se basaba en sospecha especifica? Sí ☐ No ☑ En caso que sí: ¿qué sustancia(s) deberian incluirse en el análisis?

8 Número de muestra llevadas por el representatnte de CERES: 7 Selladas ☑ Sin sellar ☐ Números de Bolsas de muestra:
Número de muestra que se quedan con el operador: 4 Selladas ☑ Sin sellar ☐ Números de Bolsas de muestra:

9 Más comentarios: [illegible] [redacted] [illegible]

(Favor siempre incluir un croquis en página 2!)

[redacted]

Firma CERES

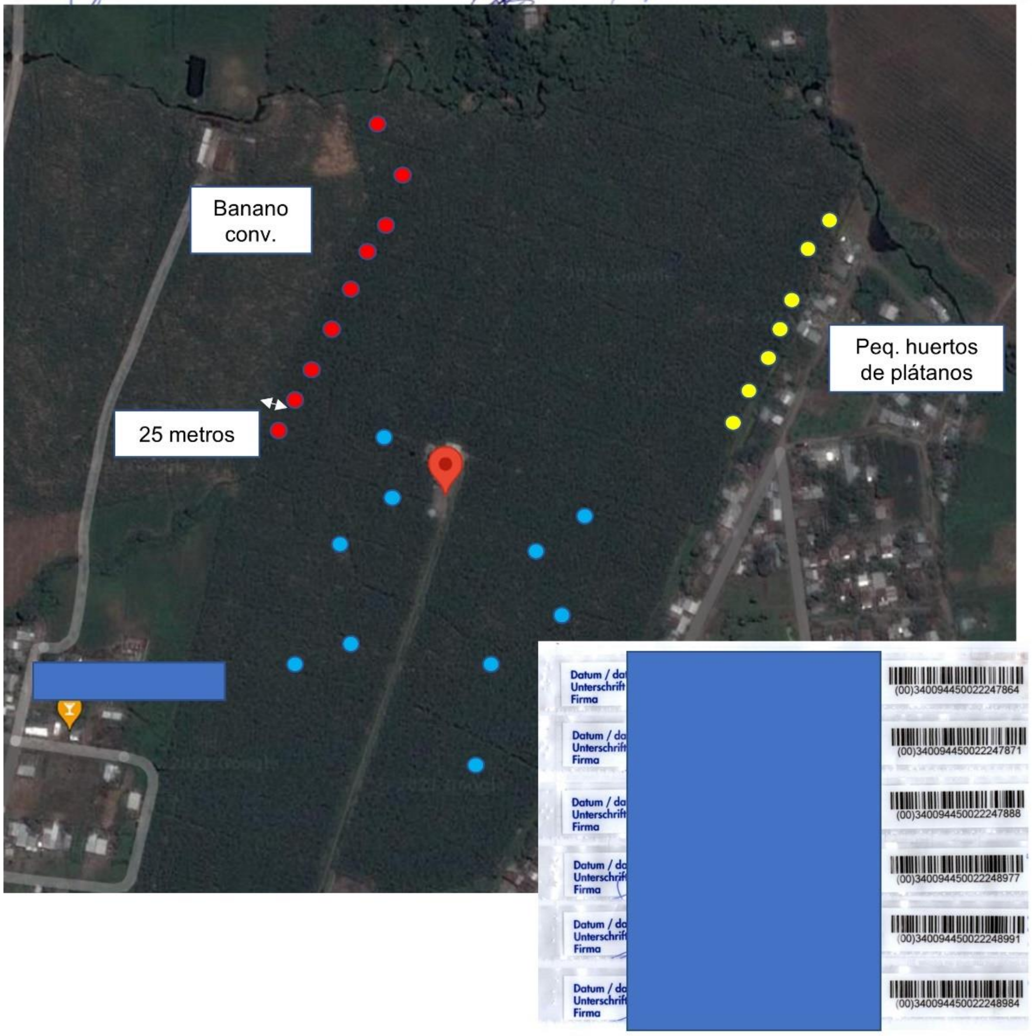

Supplementary Fig. 8: Sampling record, corresponding sampling map and bar codes with signatures on the sampling bags, from a 31 ha banana farm in Ecuador. Residues derived from aerial spray-drift were found in the NW corner of the farm (sample 1, represented by red circles), but zero residues in the centre (blue circles) and on the Eastern border (yellow circles). Distance from the conventional farm border to the other sampling areas is between 100 and 400 m. See also Figure 4b, showing the test results from this farm. Map: Google Map, version 10.49.3, https://www.google.com.ec/maps/

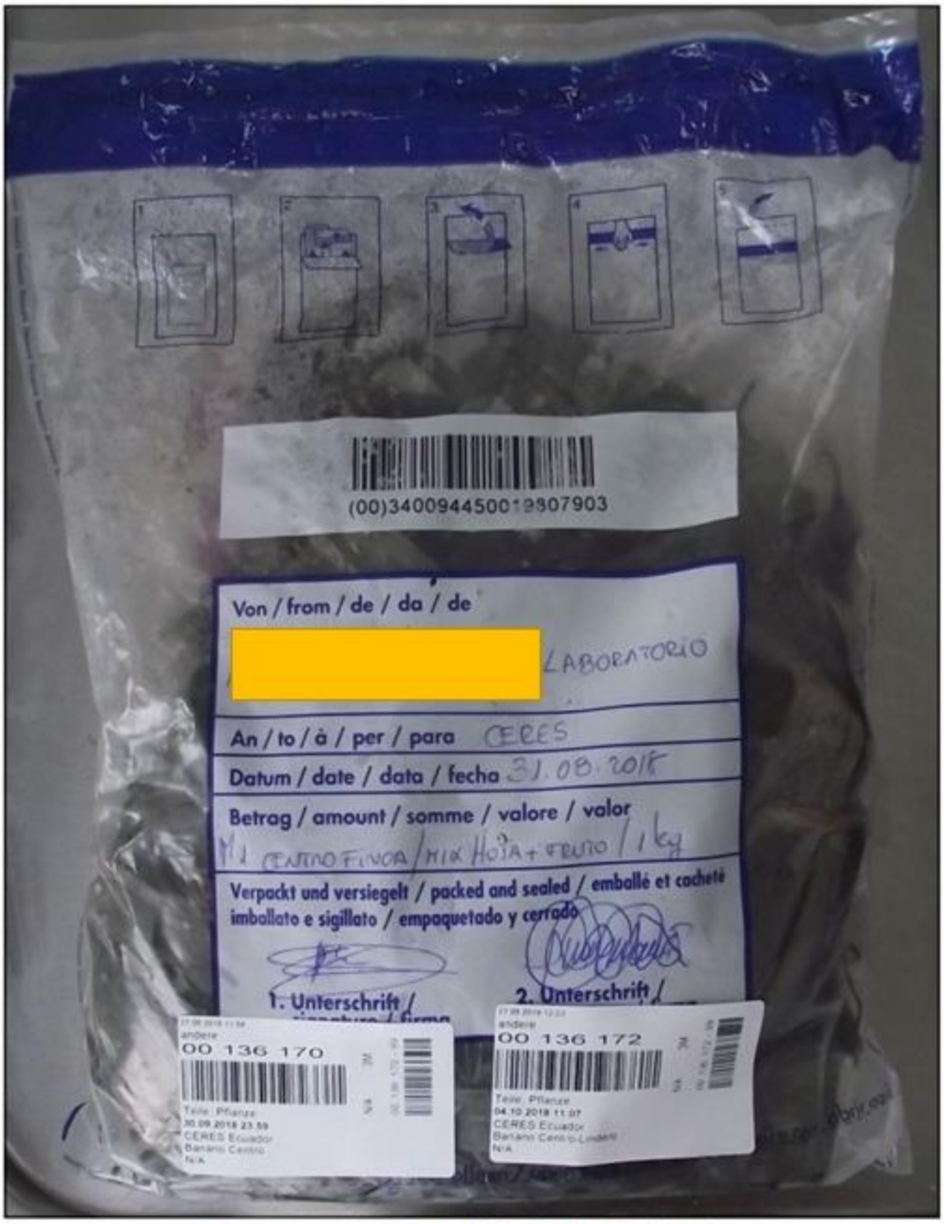

Proben Nummer: <<388-2018-00136170>>

Supplementary Fig. 9: Sealed and signed sample bag with banana leaves. For confidentiality reasons, the farm name is hidden by the orange square. Each sample bag carries a unique number and bar code. Upon receipt, the laboratory assigns its internal lot number and bar code to the sample, and takes a picture of the intact bag, before opening it. The picture is then attached to the test report.

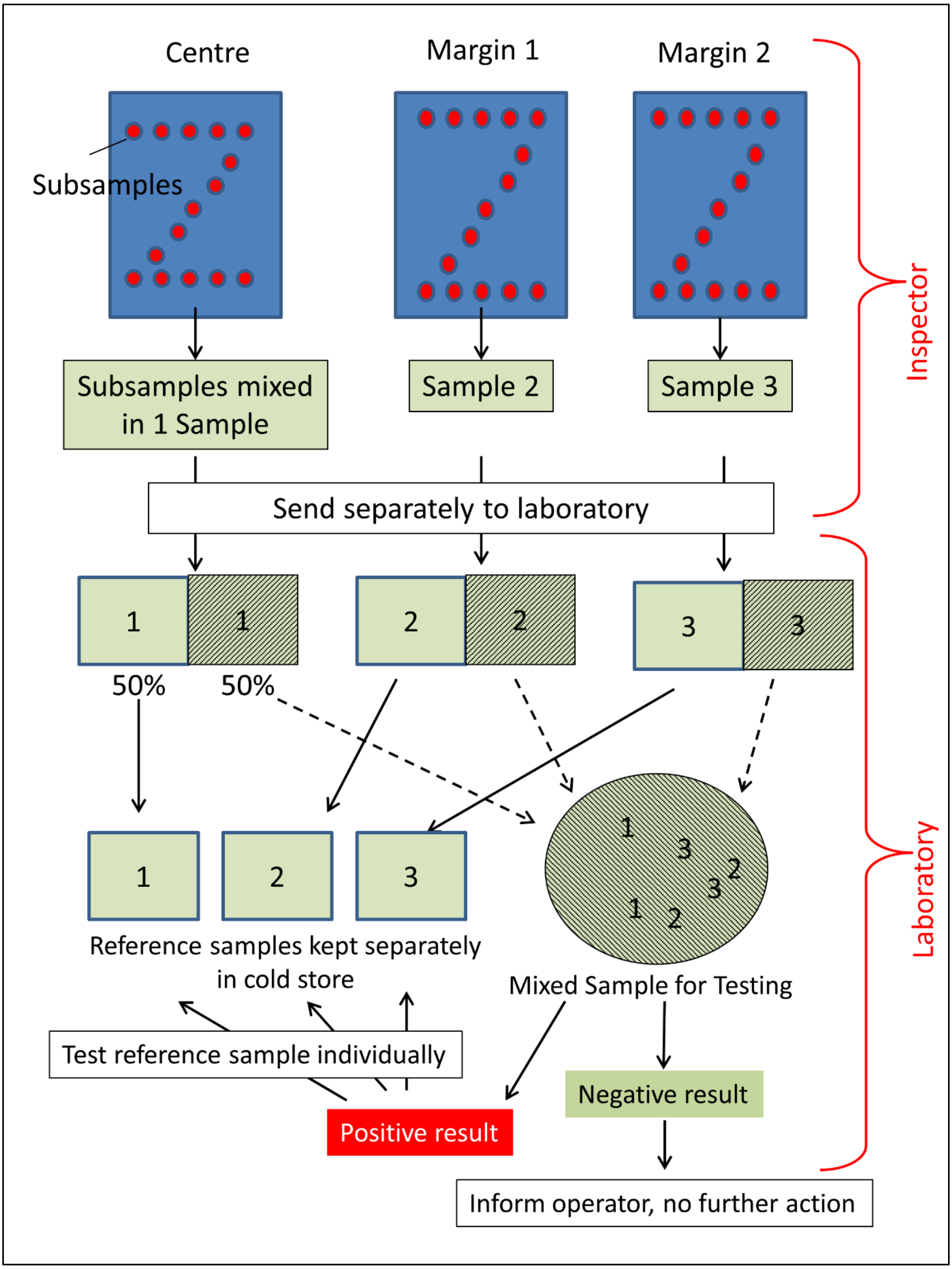

Supplementary Fig. 10: Procedure from the CERES quality manual for testing mixed samples as a first step, and individual samples in case of positive results as a second step.

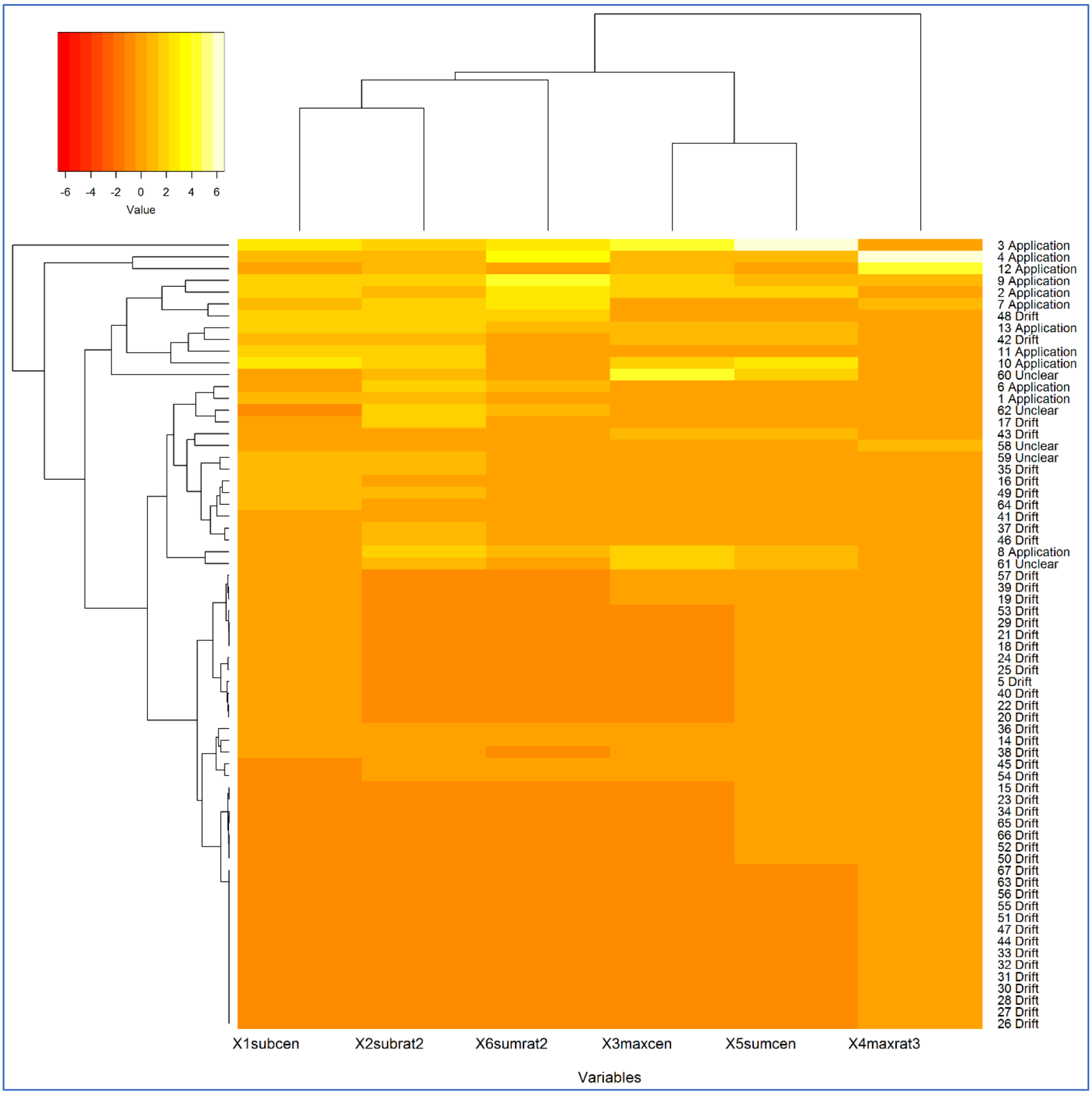

Supplementary Fig. 11: Heatmap of six variables from 67 farms. The samples appear on the heatmap according to the original classification. The “application” farms are grouped at the top of plot, the "drift" farms below. The clustering of farms is visualized using a dendrogram based on the Unweighted Pair Group Method with Arithmetic means (UPGMA).

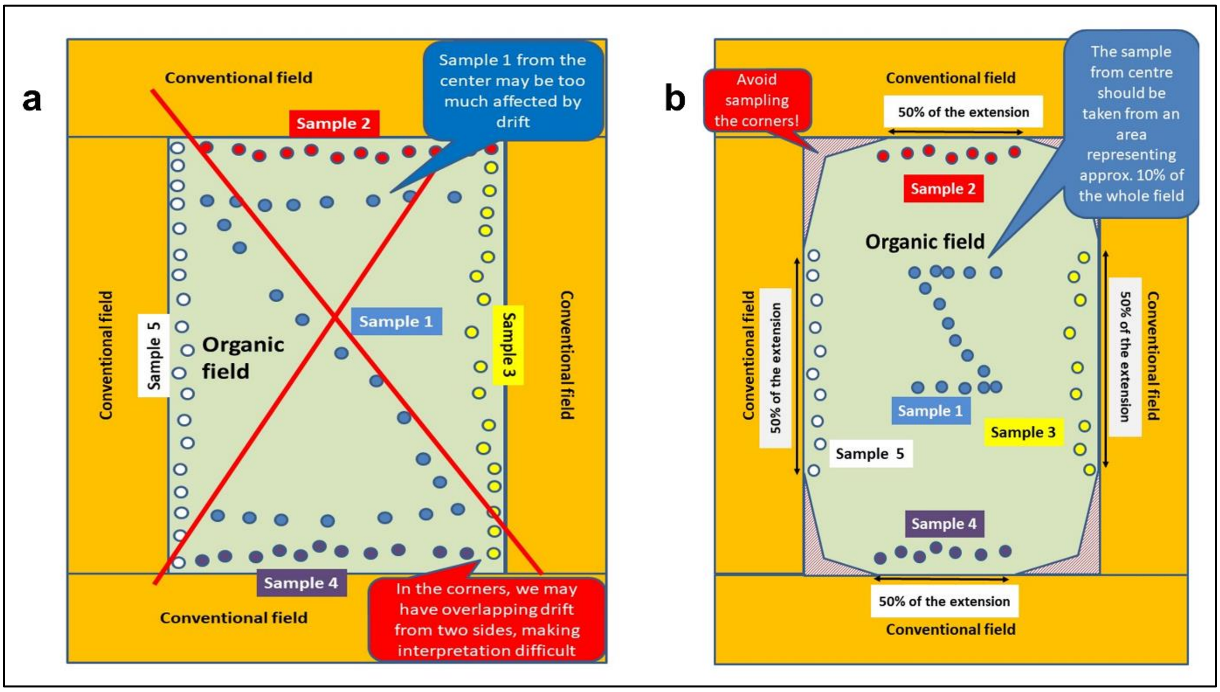

Supplementary Fig. 12: Amended work instruction from the CERES quality manual for sampling from organic fields surrounded by conventional fields, from which spray drift may originate: (a) what should be avoided, and (b) what should be done instead. This is an improvement of the procedure described in Supplementary Fig. 5(c), which was introduced after finding that inspectors were sometimes taking both centre and margin samples from too large areas, leading to results, which were difficult to interpret.

Supplementary Table 1: Fictitious example to demonstrate how the mean cumulative pesticide load per sample (MCPL) is computed.

	Sample 1 (mg/kg)	Sample 2 (mg/kg)	Sample 3 (mg/kg)	Total (mg/kg)
Acetamiprid	0.00	0.02	1.60	**1.62**
Boscalid	0.00	0.00	0.05	**0.05**
Glyphosate	0.00	0.10	0.00	**0.10**
Malathion	0.00	0.00	0.01	**0.01**
**Total**	**0.00**	**0.12**	**1.66**	**1.78**
		**MCPL:**	**1.78 / 3 ≈**	**0.593**

Supplementary Table 2: Pesticide residues in organic food sampled and tested by the USDA Pesticide Data Program (PDP) from 2013 through 2019, total and for single commodities. The latter are ranked by MCPL (column G, see Supplementary Table 1); only the 25 highest ranking commodities are listed. Heavy cream, ranking number nine, was excluded, because residues in products of animal origin are normally not linked to recent pesticide use (see Supplementary Fig. 1). Also commodities with a total of less than ten samples were omitted. The PDP selects a different set of commodities every year. Therefore, comparability across years is limited, but comparability for each commodity is given. Most commodities are repeated over two years, some over three years (in brackets after each commodity). Pesticide substances covered by the screening are standardised for all participating laboratories in every year, but the number of substances has increased steadily from 479 in 2013 to 596 in 2019.

**Commodity (Years of sampling and testing)**	**Samples**	**% of all samples[1)]**	**% samples w. res. >LOQ[2)]**	**% samples w. res. >NOP tol.[3)]**	**Cum. ∑ (mg)[4)]**	**MCPL (mg/kg)[5)]**	**Samples[6)]**		**MCPL (mg/kg)[7)]**	
							**dom.**	**imp.**	**dom.**	**imp.**
**A**	**B**	**C**	**D**	**E**	**F**	**G**	**H**	**I**	**J**	**K**
**Total:[8)]**	**3,710**	**100%**	**30.9%**	**9.8%**	**261.2**	**0.070**	**2,832**	**863**	****0.079**	**0.032**

Commodity (Years of sampling and testing)	Sam-ples	% of all sam-ples[1]	% sam-ples w. res. >LOQ[2]	% sam-ples w. res. >NOP tol.[3]	Cum. ∑ (mg)[4]	MCPL (mg/kg)[5]	Samples[6]		MCPL (mg/kg)[7]	
							dom.	imp.	dom.	imp.
A	B	C	D	E	F	G	H	I	J	K
Spinach (2015/16)	120	3.2%	81%	24.2%	111.7	0.931	104	16	*1.064	0.067
Basil (2019)	71	1.9%	89%	29.6%	60.7	0.854	51	20	0.992	0.503
Kale Greens (2017/18)	175	4.7%	79%	14.9%	28.7	0.164	170	5		
Mustard Greens (2019)	62	1.7%	37%	11.3%	8.0	0.129	62	0		
Potatoes (2015/16)	40	1.1%	100%	2.5%	5.08	0.127	37	3		
Snap Peas (2017/18)	19	0.5%	47%	31.6%	2.2	0.115	4	15		
Cherries fr.[9] (2014-16)	68	1.8%	100%	79.3%	5.41	0.108	29	39	0.027	***0.143
Peaches (2013-15)	41	1.1%	51%	17.1%	4.1	0.101	39	2		
Sweet bell peppers (2019)	12	0.3%	25%	8.3%	0.68	0.057	6	6		
Broccoli (2013/14)	37	1.0%	14%	5.4%	2.07	0.056	36	1		
Cilantro (2018/19)	24	0.6%	88%	4.2%	0.93	0.039	24	0		
Sweet potatoes (2016-18)	67	1.8%	15%	23.8%	2.58	0.039	21	0		
Honey (2017)	41	1.1%	15%	7.3%	1.58	0.038	4	37		
Apple sauce (2016/17)	58	1.6%	28%	1.7%	2.17	0.037	48	10	0.030	*0.078
Raisins (2018)	86	2.3%	99%	2.3%	2.93	0.034	60	26	0.036	0.029
Nectarines (2013-15)	40	1.1%	83%	42.5%	1.28	0.032	40	0		
Cucumbers (2015/17)	50	1.3%	36%	20.0%	1.55	0.031	15	33	0.048	0.024
Pears (2015)	66	1.8%	38%	6.1%	1.99	0.030	35	31	**0.048	0.010
Mangoes (2017/18)	30	0.8%	33%	6.7%	0.88	0.029	6	24		
Strawberries (2014-16)	69	1.9%	28%	8.7%	1.73	0.025	56	13	0.030	0.004
Cranberries canned ('18)	30	0.8%	10%	1.7%	0.73	0.024	30	0		
Grapes (2015/16)	42	1.1%	24%	9.5%	0.91	0.022	35	7		
Raspberries (2013)	55	1.5%	42%	12.7%	0.97	0.018	38	17	*0.023	0.005
Strawb. fr. [9] (2018/19)	86	2.3%	51%	1.2%	1.38	0.016	10	75	0.004	0.010
Spinach fr. [9] (2018/19)	32	0.9%	38%	0.0%	0.44	0.014	18	14	0.024	0.0005
**Corrected total MCPL** (mean of individual MCPLs, assuming that every commodity would have been sampled with the same frequency):[10]						0.041			0.042	0.020

1) Percent of the total number of organic samples taken by the PDP from 2013 to 2019 (3,710 samples)
2) Percent of samples with residues above limit of quantification (LOQ; PDP uses "LOD" = limit of detection, which is identical)
3) Percent of samples with residues above the NOP (National Organic Program) tolerance. The NOP (§205.671) establishes that products with pesticide residues above 5% of the EPA (Environmental Protection Agency) tolerance (= maximum residue limit) must not be sold with an organic label. The EPA tolerance is different for each pesticide / commodity combination (e.g. 30 mg/kg for Azoxystrobin in potatoes; 5% would be 1.5 mg/kg), therefore the percentage in this column may be low, even when the MCPL is high. When there is no specific EPA tolerance, or the 5% would be below 0.01 mg/kg, 0.01 mg/kg are used as default tolerance (USDA uses ppm, which is identical to mg/kg).
4) Cumulative sum of all residues in all samples of each commodity.
5) Mean cumulative pesticide load per sample (column F divided by column B)
6) Number of samples of domestic (= USA) vs. imported origin. H + I do not always add up to B, because the origin of some samples was not clear.
7) The MCPL for domestic vs. imported was computed only, when at least ten samples of each origin had been tested. The higher value is highlighted in yellow. Only when the higher value is identified by an asterisk, the difference is significant (based on a one-way ANOVA, with *: p<0.1; **: p<0.05; ***: p<0.01)
8) "Total" refers to all organic samples tested by the program from 2013 to 2019, therefore the values for the commodities listed here do not add up to the totals.
9) fr. = frozen
10) The corrected total MCPL was computed for all organic samples, not only those listed here.

Supplementary Table 3: Comparability of the datasets from two laboratories used in Figure 1c for pesticide residues: (mostly) ***before*** *release to the organic market (Eurofins) and* ***on*** *the (retail and wholesale) organic market (CVUA).*

Issue	Potential other reasons for lower residues in CVUA samples	Explanation
**Time of sampling and testing**	Since products already on the market are tested later, residue dissipation might explain the lower residue level.	Both datasets refer to fresh fruits and vegetables only. The time span between testing before release to the market and sampling from the market is minimum for these products, and can therefore not explain the lower residue level in the CVUA samples.
**Scope of commodities**	The definition of "fruits" and "vegetables" in the two databases could be different.	This was indeed the case. Therefore, nuts, mushrooms, herbs, and processed fruits and vegetables were excluded from the Eurofins dataset, because CVUA does not cover these under fruits and vegetables.
**Geographic origin of samples**	Eurofins could be testing more samples from countries outside the EU	This is probably true – but this is exactly part of what is shown in Fig. 1c: when businesses send organic samples from such countries to this laboratory, the purpose is selling the product on the EU (mostly German) market. What is then tested by CVUA, has already undergone the filter process.
**Substances covered by multi-substance screening methods**	If one laboratory tests for 750 substances, while the other tests for only 400, results of the former can be expected to show higher total cumulated residues.	CVUA says that each sample was tested for 750 substances, while Eurofins tests fresh fruits and vegetables for "approximately 700 substances" (the number may slightly vary from one test to another, depending on the matrix and special customer wishes). If there is any difference because of this reason, the bias should be in favour of CVUA – but this laboratory found extremely low residues in organic produce.
**Additional single-substance tests**	Inclusion or exclusion of such tests (e.g. glyphosate, dithiocarbamates, ethylene oxide) could bias the results.	Results for glyphosate and dithiocarbamates are included in the "sum of all residues". While CVUA tests all samples for these substances, Eurofins conducts these tests only on demand by customers, meaning that, similar to above, any bias should lead to higher results in the CVUA samples. 2019 was a year when the concern about ethylene oxide (EO) residues in imported products came up in the EU food industry, and many samples were tested for this substance. Since EO residues are often high, this could have biased the overall result. EO was therefore not considered for computing the MCPL.
**Non-pesticide contaminants**	Such contaminants not originating from agriculture use (see Supplementary Table 4) might be included or not.	These substances were excluded from the cumulated sum of all residues by both laboratories. Since e.g. phosphonic acid is often found at high levels especially in organic fruits, excluding it from both datasets leads to a substantial reduction in total sum of residues.
**Number of samples**	The number of samples tested by CVUA is relatively small, as compared to Eurofins, especially for organic products. These figures might therefore not be representative.	The number of organic samples from 2019 only, is indeed quite small. However, CVUA is publishing these data every year since 2013. Adding up the samples from these seven years, the laboratory tested 868 organic fruit and 604 organic vegetable samples. The results for both organic and conventional products remained very consistent across these years. This makes the data for 2019 representative.

Supplementary Table 4: Some substances defined as "pesticides" under EU food law, but in most cases not derived from agricultural pesticide use. These substances were not considered in the comparison shown in Figure 1, nor in any other sections of our article.

Substance	Explanation

Anthraquinone	Used for denaturing seeds to protect them from birds, therefore officially considered a "pesticide". In most cases, however, anthraquinone residues in food come from exposure to smoke during post-harvest handling, or from other sources of air pollution.
Bromide	Bromide is a metabolite of the fumigant methyl bromide, therefore EU food law sets an MRL for bromide. The substance, however, is also found naturally in most plants, therefore the simple presence of bromide in food does not mean it has been fumigated.
Chlorate, perchlorate	Several herbicides are chlorate based. Residues chlorate and perchlorate in food, however, are normally derived from drinking water chlorination or from chlorine based disinfectants used for surfaces in the food industry.
Diethyltoluamide (DEET)	Insect repellent. Residues are often derived from farm workers using the substances during harvest. Sometimes, sample takers themselves contaminate the samples.
Phosphonic acid	Phosphonic acid can be a metabolite of the fungicide fosetyl-Al. It can, however, also stem from phosphonate based fertilisers. In most cases, it turns out impossible to find the origin of phosphonic acid in food. While fosetyl-Al has a very short half-life, phosphonic acid is extremely persistent in soil and plant tissues.
Phthalimide	Phthalimide can be a metabolite of the fungicide folpet. Since folpet quickly degrades to phthalimide, the metabolite is calculated back to folpet. In most cases, however, residues of this substance have to do with packaging or different forms of environmental pollution, not with folpet spraying.

Supplementary Table 5: Legal provisions in different organic government standards, concerning maximum residue limits (MRLs) for organic food, and requirements for preventing spray-drift.

<table>
<tr><th>Standard</th><th>Specific MRLs for organic products</th><th>Prevention of spray-drift</th></tr>
<tr><td>Regulation (EC) N° 834/2007 (European Union, valid until 31.12.2021)</td><td>No such limits</td><td>No provisions</td></tr>
<tr><td>Regulation (EU) N° 2018/848 (valid from 01.01.2022)</td><td>No such limits</td><td>"operators shall (…) put in place (…) measures (…) to avoid risks of contamination of organic production (…) with non-authorised (…) substances"</td></tr>
<tr><td>National Organic Program (NOP, USA)</td><td>Products with more than 5% of the EPA tolerance level are excluded from organic sale, regardless of the origin of the residues (CFR §205.671). (Supplementary Table 2, Footnote 3)</td><td rowspan="6">Establishment of buffer zones between organic and conventional fields is an essential requirement in all these organic standards</td></tr>
<tr><td>Canada Organic Regime (COR)</td><td>When residues are above 5% of the MRL, the CB must initiate an immediate investigation. Below this level, the investigation can be done during the next annual inspection.</td></tr>
<tr><td>Japanese Agricultural Standard for Organic Production (JAS)</td><td rowspan="2">Products with pesticide residues must not be sold as organic – regardless of their level and origin.</td></tr>
<tr><td>GB/T 19630 (China)</td></tr>
<tr><td>Korean Organic Regulation</td><td>Maximum 5% of MRLs established by Korean food legislation.</td></tr>
<tr><td>NPOP (National Programme for Organic Production, India)</td><td>For insecticides, there is a limit of 5% of the general MRL, for other pesticides, only the general MRLs apply.</td></tr>
</table>

Decreto Supremo 2/2016 (Chile)	No such limits	

Supplementary Table 6: Half-lives in plants, and days until reaching the residue level of 0.02 mg/kg, for some selected pesticides.

Pesticide	Type	Application rate (g/ha or ml/ha)[1)]	Expected initial concentration (mg/kg)[2)]	Half-life in plants (days)[3)]	Days until reaching 0.02 mg/kg
Boscalid	Fungicide	400	20.00	6.6	66
Penconazole	Fungicide	75	3.75	8.0	60
Acetamiprid	Insecticide	200	10.00	5.7	51
Glyphosate	Herbicide	850	42.00	4.0	44
Chlorpyrifos	Insecticide	480	24.00	4.0	34
Halosulfuron	Herbicide	37	1.85	0.8	5

1) Active ingredients only. Recommended application rates depend on type of crop and its status, as well as target pest, disease or weed. The rates in this column are just examples.
2) Initial concentration rate is calculated based on the assumption of a crop with 10 t biomass/ha and 50% of the active ingredient ending up on the crop.
3) From Fantke et al. 2014[15]

Supplementary Table 7: Description of 39 variables tested for their usability for differentiating "spray-drift" from "application". The six coloured variables were most promising in the discriminant analysis. After a classification analysis, the four variables in green remained as the best for discriminating between "application" and "drift".

N°	Variable	Explanation	Scale
**1**	**1subcen**	Number of different substances (fungicides) in the sample taken in the centre of the farm	Integer
**2**	**2subrat**	Ratio of (1) to the total number of different substances in all samples from the farm	Ratio
**2a**	**2subrat2**	Ratio of number of different substances in the centre to total number of substances in all samples from the farm, but excluding cases where the maximum in the centre was < 0.03 mg/kg	Ratio
**3**	**3maxcen**	Highest single value found in the sample from the centre	mg/kg
**4**	**4maxrat**	Ratio of (3) to the highest value in all samples from the farm	Ratio
**4a**	**4maxrat3**	Ratio of highest single value in the centre, to the highest value of the same substance in any of the border samples; excluding cases where the maximum in the centre was < 0.03 mg/kg; when the substance was found **only** in the centre, 0.001 mg/kg was used for the borders because x/0 would not yield a result	Ratio
**5**	**5sumcen**	Sum of all residues in the centre sample	mg/kg
**6**	**6sumrat**	Ratio of (5) to maximum sum of all residues among the samples from the farm	Ratio
**6a**	**6sumrat2**	Ratio of the sum of all residues in the centre to maximum sum of all residues among the samples from the farm, but excluding cases where the maximum in the centre was < 0.03 mg/kg	Ratio
**7**	**7depmi**	Ratio of the minimum relative deposit of residues, which would be expected at the distance X (using a recognized drift model), and the real relative deposit of residues	Ratio
**8 to 9**	**8dep05mi** (+ following)	As (7), but only for those cases, where the highest single value is higher than 0.05 respectively 0.1 mg/kg	Ratio
**10 to 15**	**10depa** (+ following)	As (7 to 9), but using the average (10 to 12) respectively maximum (13 to 15) of all residues instead of the minimum value	Ratio
**16**	**16rmax**	Highest ratio of residues in the centre sample, to residues in the different border samples	Ratio

N°	Variable	Explanation	Scale
**17 to 21**	**17rmax05** (+ *following*)	As (16), but only for those cases, where the highest single value is higher than 0.05 respectively 0.1; 0.3; 0.5; 1 mg/kg	Ratio
**22**	**22onc**	Number of substances, which were found only in the centre sample	Integer
**23 to 24**	**23onc025** (+ *following*)	As (22), but only for those cases, where the centre value is higher than 0.025 respectively 0.05 mg/kg	Integer
**25**	**25>100**	Number of substances, for which the value in the centre is higher than the highest value from the borders	Integer
**26 to 28**	**26>80** (+ *following*)	As (25), but with 80% respectively 60% and 40% of the highest value from the border, instead of 100%	Integer
**29 to 36**	**29>10005**	As (25) to (28), but only for those cases, where the highest single value is higher than 0.05 (29 to 32) respectively 0.1 mg/kg (33 to 36)	Integer

Supplementary Table 8: Expected vs. real concentration of penconazole in an oil-bearing rose leaf sample from Bulgaria. The closest subsample was taken at 200 m distance from the claimed source of spray-drift, the average distance of the sampling points was 400 m. A conventional neighbour had applied penconazole four days before the sample was taken from the organic field. Assumptions: (a) Application rate 75 g active ingredient/ha, (b) Rose plant biomass 12 t/ha, (c) 50% of the substance end up on the crop. This would lead to an initial concentration of 3.1 mg/kg on the field from which the pesticide drift supposedly originated. (d) Half-life of penconazole is assumed to be 8 days (Fantke et al. 2001). (e) Expected concentrations are based on Equation 1. (f) Heavy wind increased drift effects by a factor 3, using approximations from APVMA[33]. The wind speed of 11 – 13 m/s during spraying by the neighbour, however, is a claim made by the farmer, which is not really plausible.

Distance	Expected concentrations (mg/kg)				% of what was actually found (0.62 mg/kg)
	without considering wind		considering 11 – 13 m/s wind		
	Initial	after 4 days	Initial	after 4 days	
200 m	0.00032	0.00023	0.00096	0.00068	0.10%
400 m	0.00006	0.00004	0.00018	0.00013	0.02%

Supplementary Table 9: Overview of 222 residue tests from centre and border samples for 25 fungicides, ranked by detected frequency among all samples. The "mean" values for individual substances include only positive findings, while the "mean" values for sum of all residues (bottom of the table) include also the samples without residues.

	Centre (n_1 = 67)				Border (n_2 = 155)				Total (n = 222)
	Find-ings	% of n_1	Mean (mg/kg)	Max (mg/kg)	Find-ings	% of n_2	Mean (mg/kg)	Max (mg/kg)	% of n
Fenpropimorph	30	44.8%	0.049	0.670	93	60.0%	0.224	3.200	55.4%
Difenoconazole	25	37.3%	0.120	0.600	94	60.6%	0.241	4.800	53.6%
Epoxiconazole	20	29.9%	0.080	0.530	90	58.1%	0.092	2.500	49.5%
Pyrimethanil	22	32.8%	0.092	1.100	70	45.2%	0.103	0.510	41.4%
Spiroxamine	20	29.9%	0.062	0.490	71	45.8%	0.115	0.740	41.0%
Tebuconazole	20	29.9%	0.054	0.410	71	45.8%	0.165	4.600	41.0%
Fenpropidin	18	26.9%	0.164	1.200	70	45.2%	0.291	2.300	39.6%
Propiconazole	19	28.4%	0.081	0.480	64	41.3%	0.125	1.800	37.4%
Triadimenol	5	7.5%	0.038	0.120	26	16.8%	0.089	0.990	14.0%
Boscalid	7	10.4%	0.032	0.140	20	12.9%	0.157	1.100	12.2%
Tridemorph	7	10.4%	0.075	0.270	20	12.9%	0.115	0.380	12.2%
Chlorotalonil	1	1.5%	0.120	0.120	17	11.0%	0.215	1.900	8.1%
Fluopyram	2	3.0%	0.013	0.020	13	8.4%	0.041	0.110	6.8%
Flutriafol	3	4.5%	0.182	0.310	11	7.1%	0.133	1.000	6.3%

	Centre (n_1 = 67)				Border (n_2 = 155)				Total (n = 222)
	Findings	% of n_1	Mean (mg/kg)	Max (mg/kg)	Findings	% of n_2	Mean (mg/kg)	Max (mg/kg)	% of n
Thiamethozan	2	3.0%	0.016	0.016	6	3.9%	0.025	0.048	3.6%
Pyraclostrobin	1	1.5%	0.076	0.076	4	2.6%	0.021	0.027	2.3%
Azoxistrobin	1	1.5%	0.005	0.005	2	1.3%	0.028	0.051	1.4%
Trifloxystrobin	0	0%	0.000	0.000	1	0.6%	0.013	0.013	1.4%
Carbendazim	0	0%	0.000	0.000	2	1.3%	0.026	0.038	0.9%
Propamocarb	1	1.5%	0.005	0.005	1	0.6%	0.340	0.340	0.9%
Dimethomorph	0	0%	0.000	0.000	1	0.6%	0.042	0.042	0.5%
Dodine	1	1.5%	0.120	0.120	0	0%	0.000	0.000	0.5%
Metalaxyl	0	0%	0.000	0.000	1	0.6%	0.010	0.010	0.5%
Tetrahydrophthalimide	1	1.5%	0.013	0.013	0	0%	0.000	0.000	0.5%
Thiabendazol	0	0%	0.000	0.000	1	0.6%	0.011	0.011	0.5%
**Sum of all residues**			**0.250**	**3.956**			**0.783**	**9.321**	
**Samples without residues**	**14**	**20.9%**			**2**	**1.3%**			

Supplementary Table 10: One-way ANOVA between "application" and "drift" for the six most promising variables. See Supplementary Table 2 concerning the description of variables and meaning of colours.

	Mean "Application"	Variance "Application"	Mean "Drift"	Variance "Drift"	p-value
1subcen	6.14	8.44	2.33	3.93	4.470E-07
2subrat2	0.81	0.09	0.17	0.09	1.307E-09
3maxcen	0.34	0.09	0.08	0.04	3.226E-04
4maxrat2	12.27	640.63	0.10	0.04	1.205E-03
5sumcen	0.78	1.00	0.12	0.06	7.613E-05
6sumrat2	1.54	2.26	0.11	0.13	8.368E-08
